# Vitamin D supplementation and growth in urban Mongol school children: Results from two randomized clinical trials

**DOI:** 10.1371/journal.pone.0175237

**Published:** 2017-05-08

**Authors:** Davaasambuu Ganmaa, Jennifer J. Stuart, Nyamjav Sumberzul, Boldbaatar Ninjin, Edward Giovannucci, Ken Kleinman, Michael F. Holick, Walter C. Willett, Lindsay A. Frazier, Janet W. Rich-Edwards

**Affiliations:** 1 Channing Laboratory, Brigham and Women’s Hospital, Harvard Medical School, Boston, MA, United States of America; 2 Department of Nutrition, Harvard T.H. Chan School of Public Health, Boston, MA, United States of America; 3 Health Sciences University of Mongolia, Ulaanbaatar, Mongolia; 4 Department of Epidemiology, Harvard T.H. Chan School of Public Health, Boston, MA, United States of America; 5 Connors Center for Women's Health and Gender Biology, Brigham and Women's Hospital, Boston, MA, United States of America; 6 Population Medicine, Harvard Pilgrim Health Care Institute and Harvard Medical School, Boston, MA, United States of America; 7 Endocrine, Diabetes and Nutrition Section, Department of Medicine, Boston University Medical Center, Boston, MA, United States of America; 8 Department of Pediatric Oncology, Dana Farber Cancer Institute, Boston, MA, United States of America; TNO, NETHERLANDS

## Abstract

**Background:**

Symptomatic vitamin D deficiency is associated with slowed growth in children. It is unknown whether vitamin D repletion in children with asymptomatic serum vitamin D deficiency can restore normal growth.

**Objective:**

We tested the impact of vitamin D-supplementation on serum concentrations of 25-hydroxyvitamin D [25(OH)D] and short-term growth in Mongol children, with very low serum vitamin D levels in winter.

**Design:**

We conducted two randomized, double-blind, placebo-controlled trials in urban school age children without clinical signs of rickets. The Supplementation Study was a 6-month intervention with an 800 IU vitamin D_3_ supplement daily, compared with placebo, in 113 children aged 12–15 years. A second study, the Fortification Study, was a 7-week intervention with 710 ml of whole milk fortified with 300 IU vitamin D_3_ daily, compared with unfortified milk, in 235 children aged 9–11 years.

**Results:**

At winter baseline, children had low vitamin D levels, with a mean (±SD) serum 25-hydroxyvitamin D [25(OH)D] concentration of 7.3 (±3.9) ng/ml in the Supplementation Study and 7.5 (±3.8) ng/ml in the Fortification Study. The serum levels increased in both vitamin D groups—by 19.8 (±5.1) ng/ml in the Supplementation Study, and 19.7 (±6.1) ng/ml in the Fortification Study. Multivariable analysis showed a 0.9 (±0.3 SE) cm greater increase in height in the vitamin-D treated children, compared to placebo treated children, in the 6-month Supplementation Study (p = 0.003). Although the children in the 7-week Fortification Study intervention arm grew 0.2 (±0.1) cm more, on average, than placebo children this difference was not statistically significant (p = 0.2). There were no significant effects of vitamin D supplements on differences in changes in weight or body mass index in either trial. For the Fortification Study, girls gained more weight than boys while taking vitamin D 3 (p-value for interaction = 0.03), but sex was not an effect modifier of the relationship between vitamin D3 and change in either height or BMI in either trial.

**Conclusions:**

Correcting vitamin D deficiency in children with very low serum vitamin D levels using 800 IU of vitamin D3 daily for six months increased growth, at least in the short-term, whereas, in a shorter trial of 300 IU of D fortified milk daily for 7 weeks did not.

## Introduction

Adequate vitamin D is required to achieve genetic growth potential among children, though a definition of “adequate” is not well established [1,2]. Vitamin D synthesis in the skin, the only source of non-dietary vitamin D, depends on skin pigmentation and exposure to ultraviolet B light, which, in turn, depends on clothing, sunscreen and latitude of residence. Above 37°N and below 37°S, ultraviolet B radiation from the sun is too weak to induce significant cutaneous vitamin D synthesis during the winter [3,4]. Mongols are at high risk for vitamin D deficiency (defined here as <20 ng/ml) [5] because they live above 45°N and have very little access to foods containing vitamin D. Furthermore, the movement of large numbers of formerly nomadic people to the city of Ulaanbaatar has resulted in increased exposure to air pollution, which influences vitamin D status by absorbing ultraviolet B radiation responsible for cutaneous production of vitamin D [6, 7]. Vitamin D deficiency in children has been linked to linear growth retardation in the presence of rickets [8, 9]. Recent evidence suggests that VDR genotypes influence height [10]. The relation between vitamin D and linear growth, however, is less clear in children without clinical manifestations of deficiency, such as bowed legs.

To test the impact of vitamin D supplementation and fortification on serum 25(OH)D levels and, secondarily, linear growth, we conducted two randomized, double-blind, placebo-controlled trials among school age children in Mongolia. The two studies were a supplementation study and a fortification study. The Supplementation Study was a pilot study, designed as a randomized, double-blind, placebo-controlled trial to test whether 800 IU vitamin D_3_ daily for six months would improve serum 25-hydroxyvitamin D [25(OH)D] concentrations, and/or protect against infection with tuberculosis [11]. Looking at the effect of the vitamin D supplementation on growth was a secondary aim. The Fortification Study was a cluster-randomized, classroom-based trial designed to test the effects of vitamin D_3_ on 25(OH)D serum concentrations and, secondarily, growth and development among children 9 to 11 years of age [12]. We examined changes in height, weight, and body mass index (BMI) among children randomized to receive vitamin D_3_ or placebo. We hypothesized that children randomized to vitamin D_3_ supplements or vitamin D_3_-fortified milk would demonstrate more linear growth than children receiving placebo.

## Materials and methods

### Study design and participants

**Fig 1:**
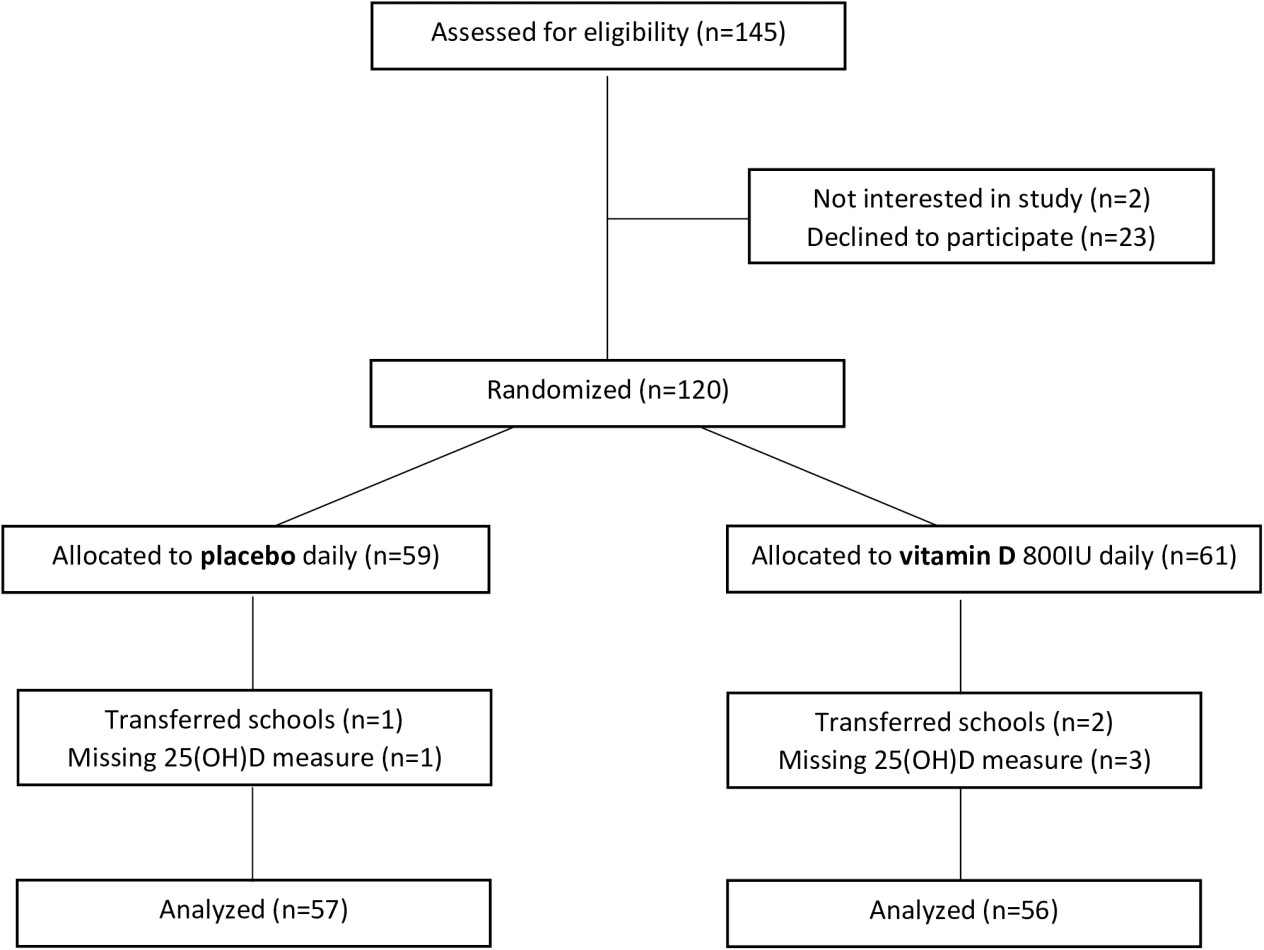
Participant flow (Supplementation Study).

The Supplementation Study enrolled children, 12 through 15 years of age, residing in Ulaanbaatar, and attending public school #65. Of the 145 children invited to the study, 120 (83%) chose to participate and were randomized. We excluded 3 children who were lost to follow-up when they transferred schools and 4 children who missed their blood draw, leaving 113 children whose results could be analyzed (Fig 1).

**Fig 2:**
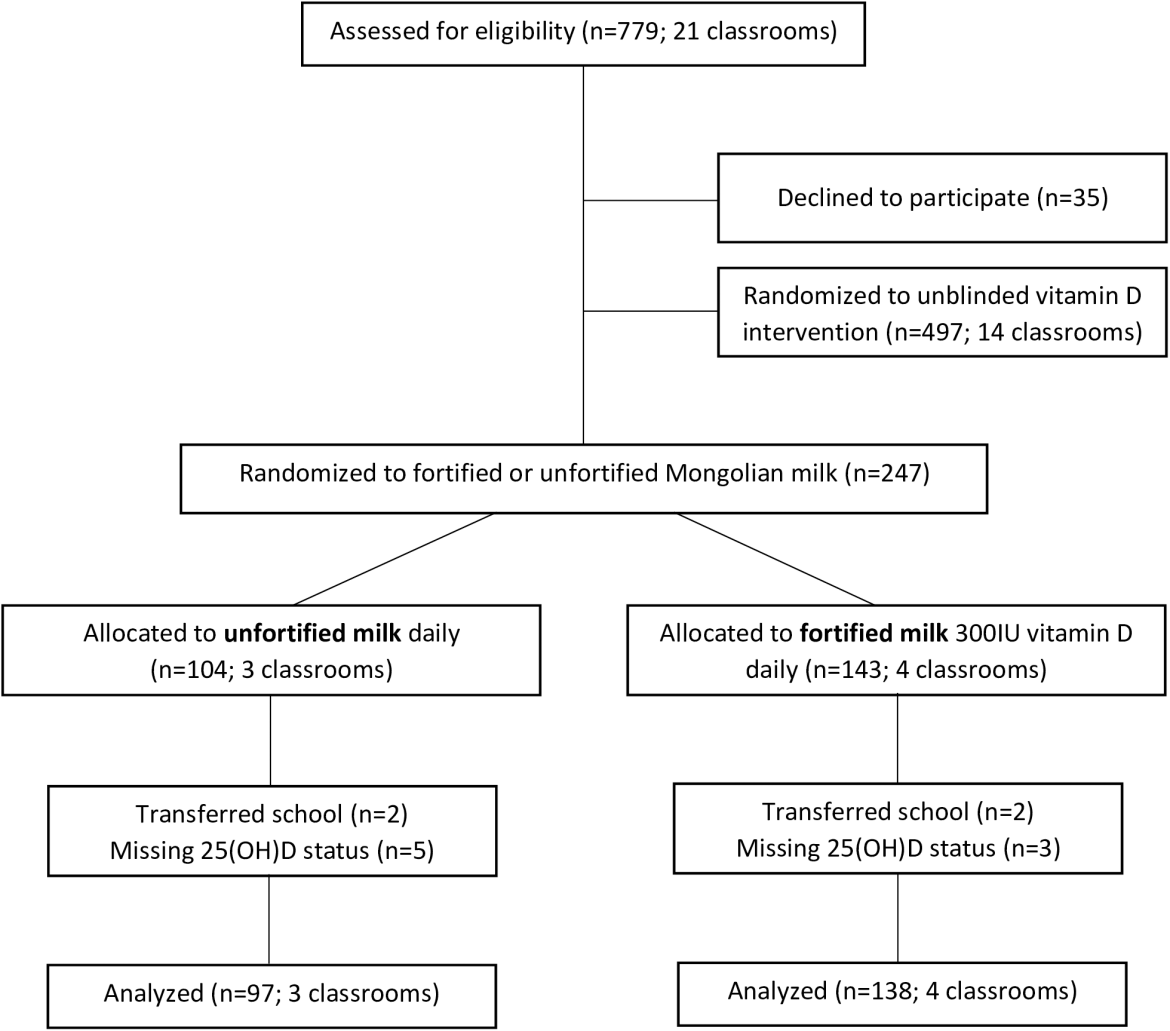
Participant flow (Fortification Study: Because we randomized classrooms, we show the number of classrooms as well as the number of students).

For the Fortification Study two public schools (#62 and #65) in Ulaanbaatar were selected because their sociodemographic profiles were similar. Children were eligible if they had no known allergy to milk. We randomized 21 third and fourth grade classrooms in each of the two schools; 744 of the 779 children in these classrooms (96%) agreed to participate. Among 247 children randomized, 4 children transferred schools and were lost to follow-up and 8 children had missing 25(OH)D values, leaving 235 children in the analysis (Fig 2). The authors confirm that all ongoing and related trials for this intervention are registered.

Both trials were registered with ClinicalTrials.gov and were given numbers NCT01244204 (Supplementation Study) and NCT00886379 (Fortification Study).

### Procedures and interventions

The Supplementation Study participants were randomly assigned to a daily tablet of 800 IU vitamin D_3_ mixed in solid matrix or an identical looking placebo. The Tishcon Corporation of Salisbury, MD, USA prepared the active and placebo tablets. After obtaining written informed assent from the children and written informed consent from their parents, eligible children were randomly assigned to either the vitamin D or placebo arm. Research assistants, school doctors, research nurses, and participants and their families were unaware of group assignments. The intervention was continued for six months, between November 2009 and May 2010.

Classrooms were randomized to one of six intervention arms in the Fortification Study. (Classrooms were the unit of randomization because some intervention arms were tablet-based and some milk-based; the children’s preference for milk over tablets would have created problems if children were randomized within classrooms). We consider here two of the six study arms—children getting unfortified Mongolian milk (n = 97) and those getting vitamin D3- fortified Mongolian milk (n = 138) as they provide a contrast in vitamin D intake using the same matrix (i.e. controlled for other nutrients) and because the students in the other four arms were not blinded to the intervention. In each of the two arms of the Fortification Study included in this analysis, children received three glasses, totaling 710 ml daily, of pasteurized, unfortified, Mongolian cow’s milk (which contains only trace amounts of vitamin D_3_) or the same milk fortified with a vitamin D_3_ premix (supplied by Cargill Inc, of Minneapolis, MN, USA). The fortified milk was formulated to deliver 100 IU vitamin D_3_ per 236 ml (8 ounce) glass, which is the standard fortification goal for milk in the United States. Both kinds of milk were packaged identically, by a local distributor, Gum Co. Ltd. All participants, their families, and study staff in the Fortification Study were kept blinded as to which milk was fortified and which not. The intervention continued for seven weeks between January and March 2009.

### Compliance

The Supplementation Study participants reported their intake of study tablets in diaries provided by the study. Tablets were given to each student each day during the school week. A trained monitor, on the staff of the school, recorded the intake on a daily basis, and reported weekly to study personnel. During holidays and weekends, participating children took the supplement or placebo at home, under parental supervision. For the Fortification Study, school staff and monitors distributed the glasses of milk, and recorded attendance and compliance, daily during the school week. Milk was not provided for holidays or weekends.

### Anthropometry

Height and weight were measured at baseline and at the end of both studies. For both studies, the height and other anthropometric measurements were conducted by trained staff members using calibrated methods. Trained research assistants weighed the children, shoeless on electronic scales. Standing height was measured shoeless, with backs against a vertical surface using a level, right-angled rod brought to the crown of the head [13]. Students removed hair ornaments and stood straight with feet flat against the base of the stadiometer and head in the Frankfort plane. Height was measured to the nearest 0.1 cm. The anthropometry form allowed the research assistants to record measurements immediately and to document any concerns about the accuracy of the height measure, such as poor positioning. BMI was calculated as weight (kg) divided by height squared (m^2^). For the Fortification Study, stages of pubertal development were self-assessed; children reported their Tanner maturation stage in 5 categories of pubic hair and breast (girls only) development, according to validated methods outlined by Marshall and Tanner (1970). As we observed only two female students with Tanner breast stage higher than 2, we combined stages 2–5 in the analysis.

### Blood samples

Professional phlebotomists drew 8 ml blood samples at baseline and at the conclusion of each intervention. The blood samples were cooled immediately, before transportation to the laboratory, where they were centrifuged, separated into aliquots, and frozen to -40°C before being shipped to the United States for serum 25(OH)D measurement.

### 25(OH)D analysis

The Supplementation Study samples were measured by the LIAISON 25(OH)D Vitamin D TOTAL Assay using chemoluminescence immunoassay (CLIA) technology (DiaSorin Inc, US) to quantitatively determine the concentration of 25(OH)D in the laboratory of Scott T. Weiss (Channing Systems Genetics and Genomics, Boston, MA) that have been validated by the CDC, according to DEQAS (Vitamin D External Quality Assessment Scheme). The Fortification Study samples were measured by liquid chromatography-tandem mass spectrometry through a turbulent flow LC system (Cohesive Technologies, Franklin, MA), followed by traditional laminar flow chromatography in the laboratory of Michael F. Holick (Boston University Medical Center, Boston, MA). The analysis was performed using a TSQ Quantum Ultra triple mass-spectrometer (Thermo Finnigan Corporation, San Jose, CA) and has been validated by the CDC, using both DEQAS (Vitamin D External Quality Assessment Scheme) and National Institute of Science and Technology (NIST) standards.

### Ethics statements

Study protocols were approved by the Institutional Review Board of the Mongolian Ministry of Health, the National University of Mongolia, and the Harvard School of Public Health. Written parental consent and written child assent were obtained prior to enrollment in both studies.

### Sample size and power calculation

Both the Supplementation Study and Fortification Study were designed primarily to measure change in 25(OH)D levels in response to study intervention. Neither study’s sample size was chosen with the aim of demonstrating differences in growth between study arms. Demonstrating growth differences were secondary aims in both the studies. However, in post hoc analysis performed using a two tailed t test to compare the arms we had 80% power to detect a difference in height change of +/- 0.91 with alpha = 0.05 for the Supplementation Study. In the Fortification Study, using the same power and alpha as we did in the Supplementation Study, the smallest detectable difference in height change between the groups was +/-0.19

### Statistical analysis

We conducted an “intention-to-treat” analysis, including all enrolled subjects, whether they were compliant or not. Our outcomes of interest were: change in weight, height, and BMI. Descriptive statistics for continuous variables were summarized as means ± standard deviations (SD) and categorical variables were summarized by proportions; these are contrasted with Student’s t-tests and chi-square tests, respectively. Modeling of the mean difference in growth parameter change between study arms required a different analytic approach for each of the two studies (Supplementation Study and Fortification Study). For the Supplementation Study we used multivariable linear regression models to compare the change in outcome between baseline and follow-up in the vitamin D3, as compared to the placebo group, an adjusted difference in differences approach. As the Fortification Study employed a cluster-randomized design, we used linear mixed effects regression models, including a random effect for classroom. The difference in differences is represented by the intervention arm’s regression coefficient.

Potential confounding variables were included in the final models if their inclusion satisfied our *a priori* criterion of a greater than 10% change in the intervention assignment coefficient [14].

Covariates considered were school (public school #62 or #65; Fortification Study only), grade (3^rd^ or 4^th^; Fortification Study only), sex, age (continuous, centered for interpretation), self-reported breast (girls only) and pubic hair Tanner stages (Fortification Study only), and type of residence [ger (circular felt tent), apartment, or house]. As Tanner stage made no significant difference in any of the models and could be included only for the Fortification Study, it was dropped from the final models. Baseline values of height, weight, and BMI were also included in models for change in height, weight, and BMI, respectively.

Effect modification of the relationship between vitamin D_3_ intervention and growth by age, sex, and baseline 25(OH)D concentration was explored by including multiplicative cross-product interaction terms in the final models. Since sex was identified as an effect modifier in one of the study models, results are presented stratified by sex. All analyses were performed using SAS version 9.4 (SAS Institute Inc, Cary, NC).

## Results

Table 1 displays the characteristics of those assigned to receive vitamin D_3_ and those assigned to receive placebo in the Supplementation and the Fortification Studies. Children in both arms of the Supplementation Study were comparable in sex, type of residence, height, weight, BMI, and baseline 25(OH)D status Table 1. For the Fortification Study, due to the classroom-level randomization, the vitamin D-fortified arm contained a larger number of students, who were slightly older and taller at baseline, on average, than those in the unfortified arm. The groups had similar distributions of sex, type of residence, weight, BMI, and baseline 25(OH)D status. Baseline vitamin D deficiency was almost universal across the two studies with mean 25(OH)D ranging from 7.0 to 7.7 ng/ml Table 1. All participants (n = 113) of the Supplementation Study had serum 25(OH)D levels less than 20 ng/ml and 91 participants (80.5%) had levels below 10 ng/ml. In the Fortification Study, the vast majority of participants (n = 235, 98.7%) had serum 25(OH)D levels less than 20 ng/ml, while 185 participants (78.7%) had levels below 10 ng/ml.

**Table 1:** Baseline characteristics of participants by study and intervention arm.

	Supplementation Study			Fortification Study		
	Placebo	Vitamin D_3_	*p*	Placebo	Vitamin D_3_	*P*
Total subjects (N)	57	56		97	138	
Male (%)	52.6	48.2	0.3	48.4	53.6	0.4
Grade 4 (%)	—	—	—	36.0	50.7	0.02
Tanner Stage 1 (%)†	—	—	—			
Pubic Hair (boys)	—	—	—	69.6	70.3	0.9
Pubic Hair (girls)	—	—	—	75.5	54.7	0.02
Breast (girls)	—	—	—	92.0	81.3	0.1
School 65 (%)	100	100		65.9	48.5	0.01
Type of residence (%)			0.6			0.8
Ger (tent)	49.1	51.7		78.3	75.3	
Apartment	1.7	0		20.5	22.3	
House	49.1	48.2		1.0	2.1	
*Mean (SD)*						
Age (years)	13.5 (1.3)	13.2 (1.1)	0.4	9.7 (0.9)	10.0 (0.8)	0.01
Height (cm)	152.3 (8.8)	151.2 (7.8)	0.5	132.7 (5.7)	134.5 (6.6)	0.04
Weight (kg)	44.6 (9.6)	42.4 (6.5)	0.2	28.8 (4.8)	29.9 (5.4)	0.1
BMI (kg/m^2^)	19.0 (2.5)	18.4 (2.0)	0.2	16.3 (1.8)	16.4 (1.9)	0.5
25(OH)D (ng/ml)	7.4 (4.0)	7.0 (3.6)	0.6	7.2 (3.8)	7.7 (4.0)	0.4

† 2 of the 97 total placebo participants had missing data on Tanner Stage

Overall compliance was 95.6% in the Supplementation Study and 84.6% in the Fortification Study. In the Supplementation Study, the vitamin D_3_ supplement group had an average increase in 25(OH)D of 12.7 ± 5.9 ng/ml with 800 IU daily for six months while the placebo group increased by 2.1 ± 3.8 ng/ml Table 2. In the Fortification Study, the vitamin D_3_ group had an average increase in 25(OH)D of 12.0 ± 5.3 ng/ml with 300 IU per day for seven weeks while the placebo group’s average 25(OH)D concentration remained unchanged (0.2 ± 3.9 ng/ml).

**Table 2:** Change in vitamin D and growth characteristics by study and intervention arm.

	Supplementation Study			Fortification Study		
	Placebo	Vitamin D_3_	*p*	Placebo	Vitamin D_3_	*p*
Total subjects (N)	57	56		97	138	
Mean (SD) compliance	95.3 (5.8)	95.9 (4.5)	0.5	87.1 (0.1)	82.8 (0.1)	0.001
*Follow-up levels [mean (SD)]*						
Height (cm)	154.4 (8.2)	154.2 (7.5)	0.9	133.5 (5.8)	135.5 (6.7)	0.02
Weight (kg)	46.5 (9.5)	44.6 (7.6)	0.2	29.9 (5.1)	31.1 (5.7)	0.09
BMI (kg/m^2^)	19.3 (2.6)	18.6 (2.2)	0.1	16.7 (2.0)	16.9 (2.0)	0.5
25(OH)D (ng/ml)	9.6 (4.0)	19.8 (5.1)	<0.0001	7.5 (3.6)	19.7 (6.1)	<0.0001
*Mean (SD) change*						
Height (cm)	2.0 (1.7)	3.0 (1.6)	0.003	0.8 (0.5)	1.0 (0.5)	0.004
Weight (kg)	1.8 (1.9)	2.2 (1.6)	0.3	1.0 (1.0)	1.2 (0.9)	0.4
BMI (kg/m^2^)	0.3 (0.8)	0.2 (0.7)	0.6	0.4 (0.6)	0.4 (0.5)	0.9
25(OH)D (ng/ml)	2.1 (3.8)	12.7 (5.9)	<0.0001	0.2 (3.9)	12.0 (5.3)	<0.0001

In the Supplementation Study, the mean increase in stature after six months was 3.0 ± 1.6 cm in the vitamin D_3_ supplement group and 2.0 ± 1.7 cm in the placebo group Table 2; this resulted in an unadjusted difference in mean growth of 1.0 ± 0.3 cm between groups Table 3. In the Fortification Study, the mean increase in stature after seven weeks was 1.0 ± 0.5 cm in children who consumed vitamin D_3_-fortified milk compared to 0.8 ± 0.5 cm in children who consumed unfortified milk Table 2; this resulted in an unadjusted difference in mean growth between groups of 0.2 ± 0.1 cm.

**Table 3:** Mean difference (standard error, SE) in growth parameter change between study arms.

	Supplementation Study				Fortification Study			
	Supplement vs. placebo arm (SE)				Fortified vs. unfortified arm (SE)			
Growth characteristics	Model 1^(1)^	*p*	Model 2^(2)^	*p*	Model 1^(1)^	*p*	Model 2^(2)^	*p*
Height (cm)								
All	0.9 (0.3)	0.003	0.9 (0.3)	0.003	0.2 (0.1)	0.1	0.2 (0.1)	0.2
Boys ^(3)^	0.8 (0.4)	0.06	1.1 (0.4)	0.005	0.2 (0.2)	0.2	0.2 (0.2)	0.3
Girls ^(4)^	0.9 (0.4)	0.03	0.7 (0.4)	0.1	0.2 (0.1)	0.2	0.2 (0.1)	0.1
Weight (kg)								
All	0.3 (0.3)	0.3	0.3 (0.3)	0.3	0.06 (0.3)	0.8	-0.05 (0.3)	0.9
Boys	-0.05 (0.5)	0.9	0.1 (0.5)	0.8	-0.1 (0.2)	0.6	-0.2 (0.2)	0.5
Girls	0.7 (0.5)	0.2	0.4 (0.5)	0.4	0.2 (0.4)	0.6	-0.02 (0.4)	1.0
BMI (kg/m^2^)								
All	-0.06 (0.1)	0.6	-0.08 (0.1)	0.6	-0.01 (0.2)	1.0	-0.06 (0.2)	0.7
Boys	-0.2 (0.2)	0.3	-0.2 (0.2)	0.3	-0.1 (0.1)	0.3	-0.14 (0.1)	0.2
Girls	0.08 (0.2)	0.7	0.01 (0.2)	1.0	0.1 (0.2)	0.7	-0.02 (0.2)	0.9

^(1)^ Model 1: adjusted for baseline height (centered; for final height model only), baseline weight (centered; for final weight model only), baseline BMI (centered; for final BMI model only) and adjusted for correlation between classrooms as a random effect (Fortification Study only)

^(2)^Model 2: adjusted for age (centered), sex (except for stratified models), grade (for Fortification Study only), school (Fortification study only), baseline height (centered; for final height model only), baseline weight (centered; for final weight model only), baseline BMI (centered; for final BMI model only), and type of residence as fixed effects (adjusted for correlation between classrooms as a random effect for Fortification Study only)

^(3)^Number of boys: Fortification Study (n = 121), Supplementation study (n = 54)

^(4)^Number of girls: Fortification Study (n = 114), Supplementation study (n = 59)

In multivariate-adjusted models adjusted for age, sex, type of residence, and baseline growth parameters, we observed a positive effect of vitamin D_3_ supplementation on linear growth in the Supplementation Study. Height change for participants consuming vitamin D_3_ was 0.90 cm greater on average than for students in the placebo arm (p = 0.003) Table 3. However, in the Fortification Study, after adjustment for age, sex, type of residence, grade, school and baseline growth parameters, with classroom as a random effect, the positive association of vitamin D_3_ with linear growth was not statistically significant (p = 0.2). The relationships between vitamin D_3_ and weight and BMI were not statistically significant in multivariate-adjusted models for either study Table 3.

There were no meaningful interactions in any outcome model between baseline 25(OH)D status and intervention in either trial. In the Fortification Study, sex was a statistically significant modifier of the relationship between vitamin D_3_ and weight change (p = 0.03), with greater weight change with vitamin D_3_-fortification among girls compared to boys. Sex was not an effect modifier of the relationship between vitamin D_3_ and change in either height or BMI. We did not observe effect modification by age on change in height, weight, or BMI with vitamin D_3_ intervention in either trial.

Table 4 examines the association of achieved 25(OH)D level with change in height. In the Supplementation Study, all quartiles of 25(OH)D had statistically significantly greater growth than children in the first quartile; the fourth quartile grew 1.4 cm more than the first quartile (p<0.001). There was not a consistent association between achieved 25(OH)D level and growth in the Fortification Study.

**Table 4:** Change in height by quartile of vitamin D at follow-up by study.

Height change (cm)	Supplementation Study					Fortification Study					
	Model 1^(1)^		Model 2 ^(2)^			Model 1^(1)^		Model 2^(2)^		Model 3^(3)^	
	β (SE)	p	β (SE)	p		β (SE)	p	β(SE)	p	β (SE)	p
Median for each quartile of vitamin D at follow-up (ng/ml)*		Median for each quartile of vitamin D at follow-up* (ng/ml)									
11.7	0.8 (0.4)	0.05	0.9 (0.4)	0.02	10.4	0.07 (0.1)	0.5	0.1 (0.1)	0.5	0.04 (0.1)	0.7
17.3	0.8 (0.4)	0.05	0.9 (0.4)	0.03	16.8	0.2 (0.1)	0.01	0.2 (0.1)	0.01	0.2 (0.1)	0.05
23.3	1.3 (0.4)	0.002	1.4 (0.4)	<0.001	24.2	0.06 (0.1)	0.5	0.1 (0.1)	0.5	0.04 (0.1)	0.7

*Note: quartile 1 serves as the reference group across all models; for Supplementation Study the first/reference quartile median was 6.4ng/ml and for the Fortification Study 5.7 ng/ml

^(1)^ Model 1: adjusted for age (centered) and sex

^(2)^ Model 2: adjusted for age (centered), sex, grade

^(3)^ Model 3: adjusted for age (centered), sex, grade, and school

## Discussion

While linear growth was not a primary outcome in either the Supplementation or Fortification Study, these results are, to the best of our knowledge, the first randomized trial results of vitamin D3 supplementation or fortification on linear growth in a population with very low serum 25(OH)D levels. In the Supplementation Study of 12–15 year old children, conducted primarily to determine the effects of vitamin D_3_ on tuberculosis infection, children randomized to 800 IU daily of vitamin D_3_ for six months experienced nearly a centimeter greater average growth than those randomized to placebo (p = 0.003). In the Fortification Study of 9–11 year old children, 300 IU daily for seven weeks did not increase height (p = 0.2).

The differences between studies may be attributable to the different durations of the trials (six months vs. seven weeks) and/or the different daily doses of vitamin D_3_ (800 vs. 300 IU).

This finding is consistent with our previous study in Ulaanbaatar where children who drank vitamin D-fortified milk grew rapidly but experienced a drop in their BMI; in that pilot study, we could not distinguish the effect of vitamin D_3_ from other nutritional aspects of the milk provided [15]. Though the beneficial effect of vitamin D on bone health has been amply demonstrated, there are limited data available on the effect of vitamin D_3_ on linear growth. In observational studies, in which supplementation was not provided, low serum 25(OH)D has been related to slower linear growth in children [16–18]. Two small trials [19, 20] examined relatively low doses of vitamin D administered over short periods of time and in populations of children with less widespread and profound vitamin D deficiency than in our Mongolian setting. In a study in Finland, 51 healthy pre-pubertal schoolchildren were randomized to either 400 IU of vitamin D_3_ 5–7 times weekly or placebo for 13 months. Although the intervention did raise 25(OH)D levels, it did not affect height or weight [19]. In a Danish study, 20 healthy children aged 6–14 years were randomized to 600 IU of vitamin D_3_ or placebo daily in a double-blind, randomized placebo controlled cross-over study with two 4 week treatment periods. There were no differences in lower leg growth during periods on placebo as compared to periods on vitamin D3 [20]. Possibly, the Finnish and Danish studies used too low a dose, or were too short in duration to observe an effect of vitamin D on height. Our participants had very low baseline 25(OH)D levels (7ng/ml) compared to the participants in the Finnish (13ng/ml) and Danish (19ng/ml) studies and correcting such severe deficiency might be more beneficial than correcting less severe deficiency. Alternatively, the small sample size of these studies may have precluded detection of an effect. The smaller of our two studies, the Supplementation Study, was more than twice the size of any previous trial of which we are aware.

Both of our studies have several limitations. We did not formally assess dietary intake. However, the average BMI of participants, at roughly 16 kg/m^2^ for the 9–11 year olds and 19 kg/m^2^ for the 12–15 year olds, is normal, indicating absence of energy deficit [21]. The Mongol diet is unusual for an Asian country, in that it consists mainly of high quality protein from meat and other animal products. In these randomized studies, any nutrients other than vitamin D would have been evenly distributed between groups; the randomization allowed us to isolate the impact of vitamin D on growth. The seven week duration of the Fortification Study was intended to demonstrate change in 25(OH)D levels and may have been too short to show growth effects.

We were interested to note that, despite different doses (800 IU vs 300 IU) (Table 2) the impact of D3 on serum 25(OH)D was about the same in both studies, resulting in an increase in mean 25(OH)D from approximately 7 ng/ml to 20 ng/ml. While at face value it may seem surprising that such different doses achieved the same increase in 25(OH)D levels, a recent systematic review by Zitterman and colleagues indicates the importance of body weight and age in serum 25(OH)D response to a given dose of vitamin D [22]. Based on the Zimmerman’s prediction formula that incorporates age and weight, we would expect achieved 25(OH)D levels of 22 and 18 for the Supplementation Study and Fortification Study, close to the observed levels of 20 for both. Thus, differences in age and weight of the participants may explain the disparate 25(OH)D increase per IU administered between the two studies. It is also possible that differences in pubertal stage and growth tempo may have influenced the children’s 25(OH)D response to vitamin D intake. In addition to the intention-to treat analysis, we also examined change in height by quartile of achieved 25(OH)D level; while not randomized, this suggested a dose-response association of 25(OH)D level with growth in the 800 IU Supplementation Study.

While randomization was used in both studies, the cluster-randomization used in the Fortification Study resulted in some imbalance between the placebo and vitamin D3 groups in terms of school and grade. These imbalances were adjusted for in the multivariate models, however, and are unlikely to have affected results.

These studies also have a number of strengths. Both studies were placebo-controlled, blinded, randomized clinical trials with high compliance and reported serum 25(OH)D concentrations, in addition to standardized measures of height and weight. We believe that our studies are the first to assess the effect of vitamin D3 supplementation and fortification on growth of children with severe vitamin D3 deficiency.

It is unclear whether the observed differences in height between groups would be maintained over a longer term. It is possible, for example, that children in the placebo group might experience ‘catch up’ growth during the summer, when their serum 25(OH)D levels would presumably have increased. However, even in the summer, 42% of adults in a Mongolian national survey had 25(OH)D levels below 20 ng/ml; levels in Ulaanbaatar, where our studies were sited, were especially low [23]. This suggests that many children may not have the opportunity for summer catch up growth that might be achieved through vitamin D repletion.

The results of the Supplementation Study suggest a stimulatory impact of 800 IU vitamin D daily on the growth of children who had extremely low levels of 25(OH)D at baseline. The 800 IU vitamin D supplements resulted in significantly increased stature, but not increased body weight. Repleting vitamin D levels for children in areas with endemic vitamin D deficiency may enable them to reach their full height potential. Future work should examine the extent to which the impact of vitamin D intervention on height depends on dose and duration, and whether any height gains last beyond the period of intervention.

## Supporting information

S1 File CONSORT-2010-Checklist-MS-Word_PlosOne.doc. (DOC)

S2 File Supplementation Study and Fortification Study protocol.docx. (DOCX)

S1 Dataset Study dataset. (XLS)
