# Supplementary material for: Vitamin D supplementation and growth in urban Mongol school children: Results from two randomized clinical trials
Source: PLoS One. 2017 May 8;12(5):e0175237. doi: 10.1371/journal.pone.0175237 (PMC5421751; doi:10.1371/journal.pone.0175237)
Supplement: S2 File — (DOCX) [file pone.0175237.s002.docx]

**Protocol summary for Supplementation Study**

(Highlight added to indicate sections especially relevant to Vitamin D and growth PlosOne submission)

1. Background

Burden of the TB epidemic: More than 95% of the estimated TB cases and deaths due

to TB occur in developing countries. Mongolia is one of the countries with highest TB

burden in the Western Pacific region. Mongolia is an ideal setting to implement the proposed study given the high prevalence of both TB and vitamin D deficiency, and the government commitment to eradicate TB. Rickets has been a common disease of childhood in Mongolia. Mongols are at high risk for vitamin D deficiency because of their northern latitude, and reduced exposure to UV-B rays during long winter, as well as low availability of vitamin D –rich food (currently no vitamin D fortified food is available in the country). However, the prevalence has even increased over the past 10 years. In 1992, a national nutritional survey by UNICEF with the MOH found that 45% of young children across the country had skeletal abnormalities of rickets. A further survey by World Vision Mongolia and Nutrition Research Center (NRC) of the Public Health Institute found that in 1997 as many as 70% of children less than 5 years of age had one or more of the clinical signs of rickets. The registered incidence of TB had increased from 79 to 141 per 100,000 between 1990 and 2001. Despite the strong effort to control the disease, including nationwide implementation of DOTS, the incidence rate of TB in Mongolia has continued to increase in the last 5 years. It is clear that Mongolians are currently experiencing a heavy burden of vitamin D deficiency. Given the strong scientific rationale for a role of vitamin D deficiency on the incidence and clinical course of TB, and the high prevalence of hypovitamiosis D in Mongolia, the setting is ideal to conduct an intervention study of vitamin D. Although the results would be most directly generalizable to Mongolians, vitamin D deficiency is very common in many populations, including African-Americans, which may also benefit from the findings.

Vitamin D, TB, and Related Infections: Sunshine and cod liver oil both have a long association with the treatment of tuberculosis and represent the two sources of vitamin D available. Earlier report from the mid-19th century suggest that vitamin D in the form of cod liver oil was beneficial in the treatment of tuberculosis. More recent reports have associated low or disturbed serum vitamin D levels with increased susceptibility to tuberculosis and this is supported by genetic linkage studies of vitamin D receptor (VDR) gene polymorphism that indicate an association between the VDR and susceptibility or resistance to tuberculosis. In vitro and animal studies provide evidence of protective relationships between vitamin D and the risks of TB. M. tuberculosis is an intracellular pathogen that resides predominantly within macrophages. There have been many studies on the role of 1,25-D3 on the innate and adaptive immune responses. In general, 1,25-D3 acts to promote the innate immune response to microbial pathogens but to quell what might be an overzealous adaptive immune response to pathogens that prove difficult for the macrophage to handle effectively. Although 1,25-D3 has direct effects on the adaptive immune system, it also affects the ability of the innate immune system to instruct the adaptive immune response. In this instance 1,25-D3 is a potent suppressor of IL-12 production and DC differentiation. Cross-sectional studies indicate that susceptibility to tuberculosis may be increased by deficiency of vitamin D. It was reported that patients with untreated tuberculosis have lower serum level of vitamin D than healthy subjects. Epidemiological evidence suggests a link between vitamin D deficiency and TB. TB is common among Asian immigrants in the UK, and the prevalence of vitamin D deficiency among this population high. A vegetarian diet, which can predispose to vitamin D deficiency, is a strong risk factor for TB in immigrant south London Asians. Despite substantial declines in TB in the United States, blacks continued to have TB at rates eight times greater than whites. Blacks have virtually half the levels of 25(OH)D compared to whites, presumably largely due to lower synthesis in skin with greater melanin content, which blocks UV-B penetration. More epidemiological data support role for innate immunity in human tuberculosis. For instance, in racially integrated nursing homes, infection measured by tuberculin skin test (TST) conversion, occurred twice as often in black as in white individuals who were equally exposed to active TB. In accordance with this, macrophages from Afro-Americans demonstrate a relative permissiveness for intracellular growth of virulent mycobacteria. Intervention studies have examined the role of vitamin D supplements in TB and other infections. In an early intervention study of vitamin D supplements that was carried out in Egypt among tuberculous children TB, clinical improvement was more evident in vitamin D supplemented children (those taking vitamin D) as compared to controls. In another small randomized, double blind intervention study in Indonesia patients were given vitamin D at dose 0.25 mg/day for 6 weeks. One hundred percent of the vitamin D group and only 76.7% of the placebo group had sputum conversion. This difference is statistically significant (p=0.002). There were also more subjects with radiological improvement in the vitamin D group.

The popularity of nutritional supplementation and vitamin D in particular, and the high vitamin D intake from vitamin D fortified food among the patients in developed countries means that the potential extra benefit of supplements might be difficult to detect. These issues can be much more readily studied in developing countries with a high TB prevalence and where background vitamin intakes tend to be low; the results, however, would be generalizable to all persons with low vitamin intakes.

Requirement for Vitamin D: Relative Contributions from Diet and Sunlight Exposure: It is generally accepted that humans evolved from apes in tropical Africa, where full-body sun exposure was a daily occurrence. Dark skin pigmentation, due to melanin, probably evolved in equatorial regions to protect individuals from deadly skin cancers or perhaps other harmful effects of UV-B radiation. Increased skin pigmentation, however, limits the individual’s ability to produce vitamin D3. As populations migrated away from the equator, intensity and duration of sunlight exposure decreased together with skin pigmentation. It was proposed that increased skin pigmentation in humans led to the development of rickets and osteomalacia, pelvic malformations, resulting in an inadequate birth canal with increased maternal and fetal mortality during migration to more northern latitudes. This scenario provided a powerful genetic basis for increased survival of lighter pigmented individuals. Humans cannot achieve adequate amounts of vitamin D from a natural diet since there is essentially no vitamin D in foods that humans normally eat except for fatty fish. In essence, vitamin D, because of its almost total lack in foodstuffs, comes almost exclusively from sunlight, and until very recently in human history, there was no need for oral administration of the vitamin. Previous studies concentrated on how much vitamin D is required to avoid clinical states of deficiency. Available evidence in which circulating intact parathyroid hormone (PTH) and 25(OH)D were measured in adult patients indicates that secondary hyperparathyroidism occurs when serum 25(OH)D values fall below the range of 15-20 ng/mL. This range has become accepted as a critical point for the beginning of vitamin D depletion. Conversely, the range of values for serum 25(OH)D in a sun-rich environment is 54-90 ng/mL.

1. Objectives/Aims/Hypotheses

We propose to examine the following hypothesis and corresponding specific aims:

That improving vitamin D status among healthy children will enhance innate immune response and will protect against TB infection.

A. To recruit subjects, obtain consent, distribute vitamin D supplements to the children in the schools, obtain biologic specimens and to transport those specimens intact back to the United States.

B. To test the hypothesis that daily vitamin D supplementation will increase plasma levels of 25(OH)D, an excellent measure of compliance, and restore TLR-induced antimicrobial activity in monocytes/macrophages tested in vitro.

Overview: We propose a feasibility pilot study with the aim to develop a full prospective study to investigate whether supplementation of vitamin D-deficient individuals with an oral vitamin D can reduce TB infection rate. Specifically, the effect of a daily 800 IU vitamin D supplement will be examined in a randomized trial: Mongolian adolescent children will be randomized to receive a daily vitamin D supplementation and placebo from the start of the study and followed through one school year.

1. Procedure and Intervention

Study design (e.g., double-blind, placebo-controlled, parallel design):

Pilot study to develop the details of the double-blind, placebo-controlled randomized trial of vitamin D in the prevention of TB infection

**Study endpoints:** the primary endpoint for this study is feasibility: 1) recruitment and consent of subjects 2) acquisition of data: height, weight, BMI, survey information (baseline background questionnaire and questionnaire regarding respiratory infection and eczema) and blood 3) compliance with the protocol 4) feasibility of shipping serum samples to the United States without degradation 5) feasibility of cathelicidn induction assay, testing subjects with T-SPOT.TB locally and confirmation of the prevalence of positive skin test at baseline.

Secondary endpoints for this study will be the absolute levels of serum 25(OH)D in the treated and control groups at baseline. Tertiary endpoints include paired differences between serum 25(OH)D at the baseline and end of the pilot study

Primary safety endpoint (study stopping rules): We propose that the DSMB meetings by conference call to decide the “stopping rule” protocol. The treatment is safe, and no case of vitamin D toxicity has ever been documented for vitamin D supplementation below 10,000 IU/day or higher. Based on the Mayo Laboratory Guidelines for 25(OH)D, the upper limit of normal is 80 ng/mL (Mayo Clinics Reference Laboratory, Rochester, MN). Operationally, we will stop vitamin D3 supplementation when if circulating 25(OH)D has exceeded 80 ng/mL serum 25(OH)D; or [2] > 2.75 mmol/L serum calcium. In recently completed studies involving high dose vitamin D3 supplementation resulting in circulating levels of 25(OH)D exceeding 300 nmol (120 ng/mL), there has been no evidence of hypercalciuria, the first indicator of hypervitaminosis D (20) For the purposes of this trial, however, to ensure maximal safety, we have included an upper limit of circulating 25(OH)Dof 80 ng/mL, above which, supplementation will be discontinued.

Study duration: 9 months

Source of Participants: Pupils of the public school # 65 in Ulaanbaatar

Inclusion criteria: Eligible subjects will be children age 12 to 15 years inclusive in public school # 65, located in Songinokhairhan district, who are tuberculin skin test negative, who are residents of Ulaanbaatar, and whose parents have given informed consent and who accent to participate in the study.

Exclusion criteria: Individuals with pre-existing calcium, parathyroid conditions, or type I diabetes, sarcoidosis, or who require chronic diuretic therapy including calcium channel blockers, or who are cognitively impaired, will not be eligible for enrollment into the study. Any individuals with active clinical symptoms of rickets at baseline will not be randomized, but referred to treatment.

Participant recruitment: Once the classrooms are chosen within the schools, a letter will be sent home to parents explaining the study and inviting parents and children to come to one of two or three informational meetings about the study (thus keeping each meeting to 10-20 parent/child pairs) Literacy rates are high in Mongolia (95% of adults can read). Dr. Ganmaa will describe the study, answer questions and invite them to participate (perhaps with the participation of Dr. Sumberzul). At the completion of the meeting and question and answer period, parents and children will be invited into an adjacent private room, where Dr. Ganmaa will administer informed consent and assent procedures, with the school doctor as witness. We will obtain child assent (and parental permission) from all eligible participants ages 12-15.

Early withdrawal of participants: When the participants are withdrawn due to stopping rules or on volunteer basis, their data will be removed from the samples. In case of detection of TB infection, the participants will be referred to the Tuberculosis Surveillance Center, National Center for Communicable Disease for the treatment.

Description: We will use vitamin D produced by Tishcon Corporation, which has produced vitamin tablets for many NIH-funded studies including those conducted by our department among children and pregnant women. Tishcon Corp. 30 New York Avenue, Westbury, NY 11590

Tiscon is willing to work with us to calibrate the actual IUs to what is said on the label. Like the milk companies, supplement companies produce their products with an overage to ensure the minimum dose in each batch. Tishcon Corp has no direct commercial interest in the results of the project. Tishcon Corp. will have no input in the design, implementation, or outcome of the study.

Treatment regimen: Subjects will be randomly assigned to receive a daily oral dose of one of two treatments from enrollment until the end of the study: (1) vitamin D (800 IU per day), or (2) placebo every school day for total nine months. All the experimental tablets will be identical in color but number-coded. The daily dose of vitamin D (800 IU) that we will use is well under the tolerable upper limit (TUL) of 2000 IU set by the institute of Medicine for children*, and is well below the level of human toxicity. The only known cases of toxicity have involved vitamin D intake greater than 40,000 IU/day. (21). **Standing Committee on the Scientific Evaluation of Dietary Reference Intakes FaNB, Institute of Medicine: Dietary Reference Intakes for Calcium, Phosphorus, Magnesium, Vitamin D, and Fluoride. National Academy Press, Washington, D.C., 1997. Available online at <http://books.nap.edu/openbook.php?record_id=5776&page=285>

Participant compliance monitoring: Substantial consideration has been given to the issue of adherence to the vitamin D intake protocol. First, 14 days after the participant is scheduled to begin taking supplementation they will be called to assess for any symptoms of toxicity. At that time, a series of brief questions will be asked to determine if the participant is having difficulty with adherence, and barriers will be addressed. Any participants who report having difficulty will be contacted again in 3 days to assess adherence and identify any additional strategies that might enhance adherence. Second, at the beginning of each month we will contact all participants and remind them that the next month of supplementation is about to begin, and address any concerns. Finally, children will take the supplementation in the class under teacher’s supervision. We will also collect any remainder pills and count those pills. Finally, we will assess compliance by self-report.

Blinding: Placebo will be provided in tablets, identical in size and appearance to vitamin D tablets. Blinded study medication will be packaged in bottles containing tablets to provide sufficient supply for each month, with overage. All site personnel will be blinded to the treatment arm assignment.

Receiving, storage, dispensing and return: Study subjects will be instructed to return all unused study supplementation in the bottle in which it was dispensed. The study research associate will be responsible for dispensing the study supplementation to participants

Study visits including procedure/tests involved at each visit (e.g., blood test, x-rays, questionnaires)

Treatment randomization will be double blinded.

a) We will recruit 3 classrooms of 100 children, expecting a maximal drop-out/non-completion rate of 10%.

b) Children and parents will complete baseline enrollment forms that query age, gender, economic background, TB contact, medical history for exclusions, and history of BCG vaccination, vitamin and supplementation intake. As part of this proposal, we propose a feasibility study to pilot test the study questionnaires and instruments (i.e., consent forms, self-administered questionnaire on diet, and lifestyle factors) to be used in the subsequent study. Mongolia is one of the countries with the highest tuberculosis burdens in the Western Pacific region. The rapid increase of TB incidence is not only related to improved detection but is also connected with transition hardship and the increase of severe poverty in Mongolia. We believe that the lifestyle factors and nutritional status of the participants assessed by the anthropometric measurements will influence the TB infection rate. In addition to agreeing to take the supplements once a day for the period of nine months, the subjects must consent to undergo a tuberculin skin test (TST) according to national guidelines and provide two 8 ml blood samples.

c) A 8 ml of blood will be drawn and cooled immediately before transportation to the Central Scientific Research Laboratory (CSRL) for further processing for T-cell assay, and where they will aliquotted into 3 x 2 cryotubes, and frozen for vitamin D and cathelicidin assay. White cells will be extracted into a pellet and stored for future genetic analysis, with the collaboration of Mongolian colleagues and if approved by the MOH Ethical Review Board. A fasting blood sample is preferred but will not be an absolute requirement, as the study biomarkers are not appreciably influenced by fasting status.

d) Baseline and endpoint measures will include: height, skin-fold thickness, and weight. The study doctor will also assess clinical signs of rickets and vitamin D deficiency. The study procedures will occur in the local school doctor’s office in private

e) Subjects will be randomly assigned to receive a daily oral dose of one of two treatments from enrollment until the end of the study: (1) vitamin D (800 IU per day), or (2) placebo. All the experimental tablets will be identical in color but number-coded. Vitamin D supplements will be administered as table. We will use vitamin D produced by Tishcon Corporation, which has produced vitamin tablets for many NIH-funded studies including those conducted by our department among children and pregnant women. Tishcon Corp. Placebo is provided in capsules, identical in size and appearance to vitamin D tablets. Blinded study medication will be packed in the ultraviolet (UV) light resistant bottles containing capsules to provide sufficient supply for each month, with overage.

The proposed doses are safe (12, 13) but high enough so as not to miss an effect, if one existed. Clearly, 400 IU vitamin D/day is not enough to sustain adequate nutritional status for many individuals in our society (14). How much oral vitamin D should be administered, particularly in a Mongolian population residing in high northern attitude, to adequately raise the nutritional vitamin D status, is of course, one of the main focuses of our proposed study.

T-cell assay: The current TST is limited because the PPD of tuberculin cannot fully distinguish between infection with TB and history of vaccination with BCG. (According to Dr. Naranbaatar, director of TB Surveillance Center of Mongolia, 30% of Mongolian school children of this age would be tuberculin skin positive). In this study, the ability to diagnose infection with M. tuberculosis in vaccine recipients is critical. In vitro t-cell based interferon release assays use antigens that are encoded by the region of difference (RD-1) so there is no cross-reaction with the BCG vaccine, since these antigens (early secretory antigenic target-6 and culture filtrate protein 10 are absent in the BCG vaccine strains (17).

Antigen-stimulated IFN-gamma production assays are highly suitable for detecting infection; and often-utilized correlate of protective immunity to M. tuberculosis infection (18) and this assay therefore served as a second indicator of the acquired host response. Two assays are currently available as commercial kits: the T-SPOT.TB assay (Oxford Immunotec, Oxford, UK) based on the enzyme-linked immunospot (ELISPOT) principle; and QuantiFERON-TB Gold (QFT-G, Cellestis, Carnegie, Australia). We will use the T-SPOT.TB assay due to its superior ability to detect M. tuberculosis in TB-endemic areas (19). It is approved for sale in Europe, Canada and other countries is being evaluated by FDA for approval. T-cell assay will performed in CSRL under supervision of Dr. Munkhbat according to manufacturer directions. Briefly, collected blood is centrifuged, PBMC are collected and resuspended in cell culture medium. Plates are incubated for 16-20 hours after preparing plates by adding cell culture medium, the antigens and control solution. Results will be determined as “reactive” or “non reactive” to TB antigens next day either manually counting the viable cell or using ELISPOT plate reader. The assays are time dependent in that the blood samples need to be transferred to the laboratory within 12 hours.

Randomization procedure:

Follow-up evaluation: There are total four visits during the study: initial, two follow-ups, and final visit (maximum 15 minutes). A background questionnaire on medical and behavioral characteristics of participants will be administered at baseline and repeated 9 months later. At every third month, trained research assistant will visit the school, administer a questionnaire on the health of the study participant in the preceding period, and a school doctors will carry out a complete physical examination. Parameters assessed at visits will include occurrence of symptoms such as fever, cough, and change in appetite and weight. Anthropometric measurements of weight, mid-upper arm circumference, and height will be taken. In addition, the following skin-fold thickness measurements will be done: triceps, biceps, and subscapular.

Complications of study procedures: Anxiety or the pain associated with phlebotomy and Tuberculin Skin Test. The participants may feel normal anxiety or pain with the blood draw and skin pricking for the TST. There is a small possibility of bruising and smaller possibility of infection or fainting. Phlebotomy will be performed by professional phlebotomists, using standard sterile technique. TST will be performed with trained nurse, using standard sterile technique. After the blood draw, traditional tea or soup will be served, as we did in the pilot study. Drs. Ganmaa, Sumberzul, RA and the medical students will be there to assist the participant in case of fainting.Drug side effects and toxicities/device malfunctions

The major risk of the study is symptoms of hypercalcaemia and hypercalcuria, which can lead to kidney stones. Potential symptoms of hypervitaminosis D and hypercalcemia are progressive weakness, fatigue, bone pain, stiffness, anorexia, nausea, vomiting, constipation, mental retardation, and excessive urination.

Although vitamin D toxicity can occur, its likelihood at the doses being used is highly unlikely. The treatment is safe, and no case of vitamin D toxicity has ever been documented for cholecalciferol below 10,000 IU/day or higher. In addition, those with high sun exposure with minimal clothing (e.g. lifeguards can make the equivalent of 20,000 IU of vitamin D or higher in a day and achieve levels of 25(OH)D about twice as high as expected in this study), and no case of vitamin D toxicity from sun exposure has ever been reported in the medical literature. Cholecalciferol from oral intake is chemically identical to that formed from sun exposure. To assess safety among those who are eligible and enroll participants will be informed on potential symptoms of hypercalcemia and asked to call the study coordinator if he or she experiences any such symptoms. Any symptoms noted will be reported to the study physician, who will make a determination if any reported symptoms are possibly related to vitamin D toxicity. If toxicity due to hypervitaminosis D or hypercalcemia is deemed a possibility, the participant will be asked to discontinue taking the pills and a blood sample for 25(OH)D and calcium will be taken

Questionnaire: Some of the questions may be uncomfortable or embarrassing to answer.

The children will be told that they do not have to answer any questions they feel are embarrassing or uncomfortable.

The breach of confidentiality is a possible risk. Confidentiality will be maintained by numerically coding data, by disguising identifying information, and by keeping all data in locked file drawers. All information obtained from subjects will be accessible only to research staff. All staff will be trained in confidentiality procedures, and routine refreshers provided.

Safety parameters: The treatment is safe, and no case of vitamin D toxicity has ever been documented for cholecalciferol below 10,000 IU/day or higher. Based on the Mayo Laboratory Guidelines for 25(OH)D, the upper limit of normal is 80 ng/mL (Mayo Clinics Reference Laboratory, Rochester, MN). Operationally, we will stop vitamin D_3_ supplementation when if circulating 25(OH)D has exceeded 80 ng/mL serum 25(OH)D; or [2] > 2.75 mmol/L serum calcium. In recently completed studies involving high dose vitamin D_3_ supplementation resulting in circulating levels of 25(OH)D exceeding 300 nmol (120 ng/mL), there has been no evidence of hypercalciuria, the first indicator of hypervitaminosis D(20) For the purposes of this trial, however, to ensure maximal safety, we have included an upper limit of circulating 25(OH)D of 80 ng/mL, above which, supplementation will be discontinued.

Definition of adverse event and serious adverse event: The major risk of the study is symptoms of hypercalcaemia and hypervitaminosis D. To assess safety among those who are eligible and enroll participants will be informed on potential symptoms of hypercalcemia and asked to call the study coordinator if he or she experiences any such symptoms. Further, we will assess symptoms at the beginning of each month when the next month’s supply of pills is provided, and at week 2 of each month when participants are called to assess problems and adherence. Any symptoms noted will be reported to the study physician, who will make a determination if any reported symptoms are possibly related to vitamin D toxicity. If toxicity due to hypervitaminosis D or hypercalcemia is deemed a possibility, the participant will be asked to discontinue taking the pills and a blood sample for 25(OH)D and calcium will be taken. The local study doctor will determine whether further treatment is necessary.

Adverse event (AE) reporting: If a child is sick, s/he will be evaluated by the local school doctor, who will examine the symptoms. The physician will report immediately to Drs. Ganmaa and Sumberzul his/her opinion of whether the illness/symptom reported is related to the trial. Dr. Ganmaa will report to the ERB and to the HSPH HSC the details of the symptoms and the If a child is sick, s/he will be evaluated by the local school doctor, who will examine the symptoms. The physician will report immediately to Drs. Ganmaa and Sumberzul his/her opinion of whether the illness/symptom reported is related to the trial. Dr. Ganmaa will report to the ERB and to the HSPH HSC the details of the symptoms and the local doctor’s opinion of the likelihood that the adverse event is related to participation in the trial (“likely association with participation”, “unlikely association with participation”, “unknown association with participation”). A case report of the adverse event will be generated and submitted to the HSPH HSC and to the Ministry of Health ERB in Mongolia. We will communicate these events as quickly as possible.

We will report adverse events according to OHRA’s Adverse Event Reporting policy: Adverse events that are serious, unexpected and related or possibly related will be reported to the IRB within 7 calendar days from the time the investigator becomes aware of the event.  Any unexpected and study-related death will be reported to OHRA within 24 hours of the investigator’s knowledge of the event by e-mail or telephone. All non-serious adverse events that are unexpected and related or possibly related to the research will be submitted to the IRB within 21 calendar days from the time the investigator becomes aware of the event.
AE/SAE follow-up: If a child is sick, s/he will be evaluated by the local school doctor, who will grade the symptoms according to the chart with Adverse Event Grades. The physician will report immediately to Drs. Ganmaa and Sumberzul his/her opinion of whether the illness/symptom reported is related to the study. Dr. Ganmaa will report to the ERB and to the HSPH HSC the details of the symptoms and the local doctor’s opinion of the likelihood that the adverse event is related to participation in the study (“likely association with participation”, “unlikely association with participation”, “unknown association with participation”). Course of action at report of Grade 1 toxicities will be decided on a case-by-case basis. The pediatrician of the child, if s/he was not the person who did the initial evaluation, will be notified of any Grade 2 toxicities. A case report of the adverse event will be generated and submitted to the HSPH HSC and to the Ministry of Health ERB in Mongolia. We will communicate these events as quickly as possible. The only special circumstances are the time zone difference between Ulaanbaatar and Boston, which can slow communication.

Mongolia that approved through OHRP as an FWA, we also propose to convene a DSMB comprised of Mongolian and American scientists to monitor the data as it accumulates from the study. The DSMB will have the authority to stop any intervention arm if they determine that it is having an adverse impact on participant health. In particular, we will ask the DSMB to monitor vitamin D levels to determine whether is causing a clinically plausible and statistically significant adverse effect.

DSMB Schedule. We propose that the DSMB meetings by conference call to decide the “stopping rule” protocol. A detailed Data and Safety Monitoring Plan will be submitted to the ERB and the Harvard Human Subjects Committee for approval prior to the recruitment of participants. During the intervention, serum samples will be taken according to the schedule. Samples will be immediately forwarded to the Health Science University laboratory for analysis, to provide timely data to the DSMB. The study biostatistician and PI will prepare reports for the DSMB, including data on participant recruitment, retention, compliance, and adverse events. The DSMB will meet at least twice during the course of the study.

- Professor Lkhagvasuren, president of the HSUM. Dr. Lkhagvasuren and the Principal Investigator, Dr. Ganmaa, have worked together for several years on the pilot projects that have led to this application.
- Dr. Ed Nardell, Associate Professor, Harvard School of Public Health, Brigham and Women's Hospital, Division of Social Medicine and Health Inequalities. He is a pulmonologist with a special research interests in TB, including the control of multidrug resistant TB (MDR-TB) in Peru, Russia, and other high-burden countries. He is past-president of the Massachusetts Thoracic Society and the North American Region, International Union Against Tuberculosis and Lung Disease, director of TB control for the City of Boston, tuberculosis control for the city of Cambridge, medical director of tuberculosis control for the Massachusetts Department of Public Health. In 2002 he joined Partners In Health as Director of Tuberculosis Research.
- Dr. Carole Mitnic, Instructor, Program in Infectious Disease and Social Change, Department of Social Medicine, Harvard Medical School. Her primary research centers on clinical outcomes research on MDR-TB, particularly in resource-poor communities.  Dr. Mitnick's current projects involve evaluating treatment protocols using novel analytic approaches and performing clinical trials of MDR-TB.  She is also working to identify surrogate endpoints for MDR-TB therapy and to assess the effect of quality improvement initiatives on TB treatment outcomes.
- Professors Radnaabazaar, the State Research Center on Maternal and Child Health, Mongolia with research interest on vitamin D and child growth and development

Professor, Michael Holick, the Boston University, international expert on vitamin D deficiency.

Confidentiality: The participants will be identifiable only by study ID. We will not divulge data to employers or insurance companies. Confidentiality will be maintained by numerically coding data, by disguising identifying information, and by keeping all data in locked file drawers. All information obtained from subjects will be accessible only to research staff. All staff will be trained in confidentiality procedures, and routine refreshers provided.

Source documentation: Our source documentation include original documents, data, and records (clinical and office charts, laboratory notes, subjects’ evaluation checklists, copies or transcriptions, records kept at the labs involved in the study. We will maintain records of our study for five years after the study in the locked files and password-protected computers of the Harvard School of Public Health and Health Science University of Mongolia

LIST OF APPENDICES:

Letter to parents describing the study

Script for the evening meeting with parents and children explaining the study

Baseline background questionnaire and follow-up

Child Study Evaluation Form

Parent Study Evaluation Form

Food Checklist

Vitamin D Results Feedback Letter

HSPH HSC Human subjects pamphlet: Mongolian version

Consent Form

Assent Form

Excerpt from K99/R00 grant application of pilot study to NIH

1. Statistical Analysis

Prior to formal analysis, preliminary analysis will include plotting the collected data to search for data and laboratory outliers.

We will then examine the following covariates to gauge the success of randomization:

- Child age in months
- Household socioeconomic status
- Baseline height, weight, and adiposity
- Baseline vitamin D

Our main analysis tool will be a relatively simple differences-in-differences approach: we will calculate the differences between the pre-intervention and post-intervention levels within each child and then compare means between the groups. This analysis will be accomplished using a simple t-test, as each pairwise comparison of treatment arms is of independent interest. In the event of meaningful violations of t-test assumptions, we will use the familiar nonparametric Wilcoxon test for differences between two groups.

1. Sample Size and Power Calculation:

We designed the feasibility pilot to generate preliminary data for a later application. As this study is a feasibility pilot, the population is chosen to represent the characteristics of the schoolchildren we would recruit for a larger, longer study. Number of subjects are limited by the budget on one hand and on the other hand, determined by vitamin D mean and SD change based on our preliminary data. We calculated to have 80% power to detect minimum detectible difference of 2.63 ng/ml and 90% power to detect minimum detectible difference of 3.89 ng/ml in mean vitamin D between the pre- and post-intervention arms with an alpha error of 0.05. In the table below, we show the minimum detectible difference in mean vitamin D between the post-intervention arms, setting Type 1 error (alpha) to 0.05 and Type 2 error (beta) to 0.80 and 0.90. We used PASS statistical software for our calculations. We considered standard deviations in 25(OHD) levels from our pilot study data, in which we observed mean + s.d. 25(OHD) levels of 17.3 + 4.8 at the outset of the intervention and 26.5 + 4.5 at the end of the one month intervention in Ulaanbaatar. However, as we lack estimates of the expected effect of antimicrobial and cathelicidin induction assay baseline levels and degree of change with the intervention, we have refrained from generating speculative power calculations for the assay.

**Summary of changes**

**AMENDMENT #1**

We increased the number of enrolled subjects from 100 to 120, and to include children with positive tuberculin skin tests (TST). In the original protocol, we have IRB approval to enroll 100 TST negative children. In our initial screening of 120 children, we found only 86 TST negative children. Given this high baseline rate of TST positivty (28%), which had been heretofore unknown in Mongolia, we would have had to screen at least 130 children to find 100 TST negative ones. However, our screening provided the first data of this kind ever for Mongolia. The high rate of TST positivity in clinically healthy children made it clearer to us than it had been before that a TST is useful clinically in Mongolia only if it is negative, because of the extensive use of BCG vaccination in that country.

Having found so many TST positive children, we felt we had an unanticipated ethical problem. Excluding the TST positive children from the study would have labelled them as possibly infected with active TB to their families, classmates, neighbors and teachers. The psycho-social consequences of that could be disaterous. By enrolling TST positive children in the study we would be providing greater protection for our subjects.

We therefore propose to update the inclusison/exclusion criteia as follows: eligible subjects will be children ages 12 to 15 years who attend public school # 65, located in the Songino-Khairhan district of Ulaanbaatar, and whose parents have given informed consent and who themselves assent to participate in the study. Exclusion criteria will be: pre-existing disorders of calcium metabolism, type I diabetes, sarcoidosis, chronic diuretic therapy including calcium channel blockers, or cognitive impairment.

**AMENDMENT #2**

Additionally, we collected data by means of a questionnaire on respiratory infections and eczema since there is good evidence that vitamin D supplementation may prevent such conditions. If we are to collect these data, it will be vital to do so before the unblinding of subjects occurs.

**AMENDMENT #3**

In deference to issues raised by the Mongolian IRB, we changed number of blood draws from two times to two to three times as it was considered informative to have sub-samples of serum vitamin D levels in mid-winter (January). This change has been incorporated into consent and assent forms.

**Protocol summary for Fortification Study**

(Highlight added to indicate sections especially relevant to Vitamin D and growth PlosOne submission)

1. Background

Vitamin D deficiency: Mongolians are at high risk for vitamin D deficiency because of their northern latitude and reduced exposure to UV-B rays during the winter months. There is also low availability of vitamin-D rich foods. Although one local milk producer, Gum, has begun to produce vitamin D fortified milk, the vast majority of milk sold in Mongolia is not vitamin D fortified. In a 1992 survey conducted by UNICEF and the Mongolian Ministry of Health and Social Welfare, 44% of children less than age 5 had clinical signs of rickets (either craniotabes and/or bowed legs or knocked knees) (UNICEF). A supplementation program was instituted for children under 2 years of age, but a repeat survey in 1997 conducted by the Nutrition Research Center and World Vision Mongolia demonstrated that 72.3% of children under age 5 exhibited at least one of nine clinical sign of rickets. The prevalence of rickets was highest in the capital city, Ulaanbaatar, which has experienced rapid growth over the decade since Soviet withdrawal, as harsh economic conditions in the countryside forced migration to the city. In addition to the established and recognized impact of early childhood vitamin D deficiency on the prevalence of rickets, low vitamin D levels throughout life have recently been associated with infectious disease (including influenza and tuberculosis); have been shown to exacerbate asthma, osteoporosis, and skin diseases such as eczema; and may increase the risk for colon, prostate and breast cancers. Among schoolchildren, low vitamin D levels may also affect bone development and growth, especially during the rapid peripubertal growth spurt.

There are few data on vitamin D levels of school-age children. In May 2005, we measured levels of 25(OH)D in an Ulaanbaatar classroom, and found that 76% of children had 25(OH)D levels <50 nmol/L (<20 ng/ml) at the start of the intervention. In one month of drinking 710 ml daily vitamin D fortified American milk, the children’s vitamin D levels were markedly improved: 93% were > 50 nmol/L. (The level of ‘normal’ 25(OH)D is debated; if we consider the higher threshold of 62 nmol/L (25 ng/ml), 93% of children were below normal at the start of the study and 37% at the end).(Ganmaa D, Tserendolgor U, Frazier L, Nakamoto E, Jargalsaikhan N, Rich-Edwards J. Effects of vitamin D fortified milk on vitamin D status in Mongolian school age children. Asia Pacific Journal of Clinical Nutrition 2008, in press). This feasibility study lacked a control group, and it was not designed to test alternative vitamin D repletion strategies. We propose in this new study to compare change in winter levels of plasma 25(OH)D among four groups: monthly vitamin D supplements, daily vitamin D supplements, daily fortified milk, and daily unfortified milk. Supplements may be appropriate for distribution in the countryside, where milk is consumed from family herds. It may prove easier to distribute and administer monthly supplements to children than daily supplements; however, it is unknown whether monthly supplementation is as effective as daily supplementation. Our trial will test this. We will also compare the impact of supplementation with milk fortification. The advantage of fortification is that it does not require governmental distribution; instead, it relies on consumer behavior. The other advantage is that milk also delivers calcium, which may help to raise vitamin D levels. We will test this in this trial.

Respiratory illness, asthma, and eczema: In Boston, Dr. Camargo recently demonstrated that winter-related eczema symptoms were ameliorated by vitamin D supplementation. (Sidbury R, Sullivan AF, Thadhani RI, Camargo CA Jr. Randomized trial of vitamin D supplementation for winter-related atopic dermatitis in Boston: A pilot study. Br J Dermatol 2008; in press) We propose to test the impact of the repletion strategies on the prevalence and severity of eczema symptoms.

Dr Camargo also has demonstrated that 25OHD levels are inversely associated with risk of respiratory infection and wheezing illness in New Zealand children.(Camargo CA Jr, Ingham T, Wickens K, Thadhani RI, Silvers KM, Epton MJ, Town GI, Espinola JA, Crane J. Cord blood 25-hydroxyvitamin D levels and risk of childhood wheeze in New Zealand. Am J Respir Crit Care Med 2008; in press) We propose to test the impact of the repletion strategies on the incidence of respiratory infections, including among children with asthma.

Academic attention: Teachers in the pilot study school volunteered in conversation that they felt that the children paid better attention during the milk intervention month. If true, this is probably due to the increased calories of the intervention. If we were to document improved attention with the milk intervention, this would be useful information for the Ministry of Education’s debate regarding school lunch programs. We will ask the classroom teachers to complete an evaluation of each student’s attentiveness and behavior before and after the intervention. We would also like to pilot test a translation of the computer-based Continuous Performance Test, commonly used to measure attentive focus.

Milk, somatotropin levels, and childhood growth: Cow’s milk is known to promote childhood growth, bone development, and to increase plasma levels of IGF-1 and the ratio of IGF-1 to its binding protein, IGFBP3. In our 2005 feasibility study, we observed that children grew rapidly in height during the month-long milk feeding intervention (an average of 1.1 cm), and had marked increases in plasma levels of IGF-1, IGF-1/IGFBP3, and growth hormone (GH).( Rich-Edwards JW, Ganmaa D, Pollak M, Nakamoto EK, Willett WC, Frazier L. Milk consumption and the prepubertal somatotropic axis. Nutr J 2007; 6:28) It is not known whether the nutrient content or the endogenous bovine hormone and growth factor content of milk drives the increased growth and somatotropin hormone levels among children who drink milk. All mammalian milks contain natural hormones and growth factors produced by the mother; this is true of American milk and Mongolian milk. The milk we used in the pilot study and the milk we will use in the proposed study are from a manufacturer (Borden) who claims that their farmers do not use exogenous hormones to raise milk yield (i.e., BST or BGH). In theory, different milks may have varying hormone and growth factor content, depending on husbandry practices, processing, and fat content. We propose to test the impact of drinking U.S. cow’s milk, Mongolian cow’s milk, and a vegetable-based nutrient substitute for cow’s milk on plasma somatotropin levels and growth.

Our original research question at the time of the pilot study was that the natural estrogens in cow’s milk might increase estrogen levels in children, with a hypothetical, unproven, question of whether higher estrogen levels might be associated with risk of cancer in adulthood. In fact, in both our pilot studies in Boston and Mongolia, we observed no increase in estrogen levels of children drinking conventional American milk (see excerpt from report to the Ministry of Health in Appendix 15). Our interest at this point is in pursing the question of how fortified milk can affect vitamin D levels and growth in children.

###### Specific Aims

The goals of this two-year project are to raise national awareness in Mongolia regarding the public health impact of widespread vitamin D deficiency, to improve Mongolian capacity to monitor and redress vitamin D deficiency, to strengthen research capacity, and to provide data on supplements and food fortification as alternative strategies to prevent vitamin D deficiency. To these ends, we will convene a national conference, set up a national vitamin D laboratory, and conduct an intervention trial. Having observed rapid linear growth in Mongolian children drinking fortified milk in our pilot study, we would also like to assess the impact of milk consumption on growth and plasma somatotropin levels. The three-month intervention trial will test the impact of vitamin D fortified milk and vitamin D supplements on plasma levels of vitamin D (25(OH)D), plasma somatotropin levels, short-term growth, and respiratory infections, asthma, and eczema in Mongolian prepubertal children. Teachers will complete a Classroom Behavior Checklist for each child before and after the intervention, so that we can compare the impact of the interventions on classroom attention and behavior. We would also like to adapt and test the Continuous Performance Test (CPT) of attention in a subgroup of the students to document the (presumed increase) in attention that we expect to come from improved nutrition.

The aims of the intervention trial are:

Specific Aim 1: To measure the prevalence in winter of vitamin D deficiency among Mongolian schoolchildren and to compare the impact on plasma 25-hydroxyvitamin D (25(OH)D levels of two vitamin D supplement regimens and vitamin D fortified milk. Specifically, we hypothesize that after three months of intervention from January to March:

1. Daily vitamin D supplements of 300 IU will yield higher levels of 25(OH)D than will monthly supplements of 6000 IU.
2. Daily consumption of milk fortified with 300 IU will yield higher levels of 25(OH)D than will either supplement regimen.
3. Supplementation and fortification will each yield higher levels of 25(OH)D than consumption of unfortified milk.

Specific Aim 2: To test the impact of various types of milk on childhood plasma somatotropin levels and growth. Specifically, we hypothesize that:

1. Daily consumption of cow’s milk will yield higher levels of growth hormone, IGF-1, and IGF-1/IGFBP3 than will daily consumption of equivalent nutrients in a milk substitute.
2. Daily consumption of U.S.-produced cow’s milk will yield higher levels of these somatotropins than will daily consumption of Mongolian cow’s milk.
3. Daily consumption of cow’s milk will cause greater increases in height and sitting height than will daily consumption of a milk substitute.

(Secondary) Specific Aim 3: To measure the prevalence of winter-related eczema among Mongolian schoolchildren and to estimate the impact of vitamin D supplementation and fortification on eczema prevalence throughout the winter. Specifically, we hypothesize:

1. Children randomized to vitamin D supplements or fortified milk will have a reduction in symptoms of winter-related eczema.
2. Children randomized to vitamin D supplements or fortified milk will have fewer symptoms of winter-related eczema after three months than children randomized to consume unfortified milk

(Secondary) Specific Aim 4: To measure the prevalence of asthma among Mongolian schoolchildren and to estimate the impact of vitamin D supplementation and fortification on respiratory infections throughout the winter. Specifically, we hypothesize:

- 1. Children randomized to vitamin D supplements or fortified milk will have fewer respiratory infections after three months than children randomized to consume unfortified milk.
  2. Asthmatic children randomized to vitamin D supplements or fortified milk will have better asthma control after three months than children randomized to consume unfortified milk.

C. Study Procedures / Interventions

Study Setting: Mongolia is a landlocked country in Central Asia surrounded on the east, south and west by China and to the north by Russia. In 1921, after the Russian Revolution, it became the second Communist country in the world – the Mongolian People’s Republic. With the advent of perestroika and the collapse of the USSR, Mongolia became a democratic nation in 1990. Today, Mongolia has 3 million citizens, of whom over 800,000 reside in the capitol city, Ulaanbaatar. Mongolia enjoys a literacy rate of 95%, and has well-organized public schools that are served by state-paid local family doctors. The health care of all children is covered by national health insurance.

Study Population: There are ~710 primary schools in Ulaanbaatar, with roughly 560,000 children.

1. Recruitment: In collaboration with the Health Sciences University of Mongolia, we propose to recruit 750 girls and boys in 3rd grade from 10 Ulaanbaatar public schools. The children will be 9-11 years old.

2. School selection: School selection will consider the following factors: socioeconomic position; geographic location; school size; current lunch or supplementation program; and the interest and willingness to participate of the school’s administration and faculty. Dr. Ganmaa will travel to Mongolia in May 2008 to visit schools and talk to officials about possible participation. We will get written documentation from principals of schools who agree to participate.

3. Eligibility: Children will be eligible to participate if they report no known allergies to milk, nuts, coconut, or wheat.

Study Design

1. Six-arm randomized partially-blinded feeding intervention study to test the impact of vitamin D supplements and fortified cow’s milk on the plasma 25(OH)D, growth hormone, and IGF-1 of prepubertal children. As detailed below, the arms include: monthly vitamin D supplements (n=100); daily vitamin D supplements (n=100); daily fortified U.S. cow’s milk (n=150); daily fortified Mongolian cow’s milk (n=150); daily fortified milk substitute (n=150); and daily unfortified Mongolian milk (n=100).

2. The intervention will run for three months, from January 2009 through March 2009, to coincide with the darkest months of the year. There will be a two-week break in the intervention to coincide with the school holiday in February. (It is highly unlikely that children would leave Mongolia during this period, so there are no concerns about increased sunlight exposure over vacation.)

3. Study measures will include: height, sitting height, weight, grip strength, asthma, eczema, respiratory illness, and (at baseline only) clinical signs of rickets and Tanner stage (sexual maturity). An 8 ml blood sample will be drawn at baseline and at the conclusion of the intervention. Classroom attentiveness and behavior will be rated by the classroom teacher for each child at the beginning and end of the intervention. A subset of children will perform the Continuous Performance Test as a pilot.

Intervention

1. A series of introductory school-based meetings will be led by Dr. Ganmaa to describe the intervention study to parents and students, answer their questions, and invite them to participate. School administrators and the local school doctor will participate. (November/December 2008) Depending on availability, Drs. Sumberzul will also participate.
2. At a second meeting, children and parents will complete an eligibility interview to assess disqualifying allergies to any intervention product. For eligible and willing participants, parents will complete an informed consent form and children will complete an informed assent form. (December 2008).
3. After consenting/assenting, children and their parents will complete a questionnaire, including questions regarding family socioeconomic status, a food frequency questionnaire, and ISAAC questions regarding wheezing/asthma and eczema.( Ellwood P, Asher MI, Beasley R, Clayton TO, Stewart AW. The International Study of Asthma and Allergies in Childhood (ISAAC): Phase Three rationale and methods. Int J Tuberc Lung Dis 2005; 9: 10-16.) This constitutes the first data collection of the study; thus we consider the data collection period to last from November through the end of the January-March intervention period. During the course of the study, the children will also complete a dairy checklist to assess dairy consumption in the first week of the trial, pre-vacation, post-vacation, and in the last week of the trial.

1. At baseline and at the conclusion of the study, professional phlebotomists (who will have completed human subjects training) will draw an 8ml sample into a red top tube. Blood draws will be done in the morning (8:00 to 10:00 am) to minimize for the diurnal variability in GH and other hormone levels. At the end of the intervention, samples will be taken on a Thursday or Friday, so that milk exposure during the school week will have its full effect, and any effect of weekend diet is minimized.

Blood samples will be collected and cooled immediately before transportation to the Health Sciences University of Mongolia Laboratory, where they will be spun, aliquotted into 4 x 1ml cryotubes, and frozen. White cells (DNA) will be discarded. To guard against freezer failure, one set of aliquots will be kept in the electrical -70 freezer (with generator backup) at the HSUM laboratory, and the other set in a liquid nitrogen -80 freezer at the laboratory of Dr. Munkthaivan, with whom we collaborated for the pilot study. Assays will be run in Canada, and will require shipment by express carrier on dry ice. We accomplished this during the pilot study, and are seeking even faster methods with the cooperation of Korean Air and courier services.

1. **Trained medical student volunteers will measure height, sitting height, weight, and grip strength at both baseline and at the conclusion of the study.** Trained medical volunteers will assess Tanner stage and rickets symptoms at baseline only.
2. Trained medical student volunteers will perform a dermatologic exam to derive Investigator Global AD Assessment (IGADA) and EASI score at both baseline and at the conclusion of the study.
3. Classroom teachers will complete the Classroom Behavior Questionnaire for each child at the beginning and end of the study. For a subset of students, trained medical student volunteers will perform the Continuous Performance Test with children at baseline and at the conclusion of the study. (The computer will generate dot matrix numbers (3..6..2..8..1..10..) that will appear on the CPT screen. The child will be asked to push a response button each time they see the target stimulus preceded by a certain number appear on the screen. The task should take approximately 15 minutes.)
4. **The first week of the milk intervention will be a ‘phase-in’ period, in which 236 ml/per day of milk or milk substitute will be consumed for the first three days, followed by 472 ml/per day over the next two days**. **In the second week, the full 710 ml protocol will be initiated**. The vitamin D supplement arms will be phased in accordingly (100 IU days 1-3, 200 IU day s 4-5; 300 IU thereafter for the daily supplement arm; 5200 IU will be given for the first monthly dose for those in the monthly supplement arm. See schedule in table 1.

Table 1. Schedule of vitamin D fortification/supplementation

|  | Schooldays | Daily IU in daily supplement and fortified milk arms | Monthly IU in monthly supplement arm |
| --- | --- | --- | --- |
| January | 20 | 3 days @ 100 IU  2 days @ 200 IU  15 days @ 300 IU  (Total=5200 IU) | 2 days@ 2000  1 day @ 1200 IU  (Total=5200 IU) |
| February | 10 (half month due to vacation) | 10 days @ 300 IU  (Total=3000 IU) | 1 day @ 2000 IU  1 day @ 1000 IU  (Total=3000 IU) |
| March | 20 | 20 days @ 300 IU  (Total=6000 IU) | 3 days @ 2000 IU  (Total=6000 IU) |

1. One staff member in each school will be hired and trained as a ‘milk monitor’ to: receive supplies from the distributor; wash and boil the measuring cups, spoons, openers, and pitchers daily; prepare the milk substitute; distribute milk or supplements to individual children in the classrooms; observe that the milk or supplements are consumed; collect empty servings; and record compliance. After the initial phase-in week, children will receive 710 ml of milk each school day, as they did in the feasibility study. Children will not be pressured to finish their milk; the compliance recording is taken to document compliance for secondary analysis of amount of milk or supplements consumed. The Mongolian milk producer, Gum, will deliver the Mongolian study milks. The Project Director will deliver the U.S. milk and supplies for mixing the milk substitute. The US milk and Mongolian milks already come in sterile packaging. The milk monitors will mix the milk substitute at the school and serve the milk substitute in disposable paper cups. Once mixed for the day, the milk substitute will be stored in a refrigerator for the day. Extra milk will be discarded at the end of the day.
2. Dr. Ganmaa will monitor the mixing of the milk substitute by the milk monitors, to ensure both its safety and its standardized nutritional content, performing unannounced “spot checks” at each school to verify that procedures are being followed exactly.
3. Parents and children will complete a study evaluation form at the end of the study when we meet to present the overall study results.

Intervention Arms

Children will be randomized to receive:

1. 300 IU in 710 ml of U.S. vitamin D fortified milk on school days (n=150)
2. **300 IU in 710 ml Mongolian vitamin D fortified milk on schooldays (n=150)**
3. **710 ml of the typical unfortified Mongolian milk (n=100) These children will receive vitamin D supplements at the end of the study equivalent to the amount consumed in the fortified milk arm**.
4. 300 IU in 710 ml on schooldays of milk ‘substitute’ that has the same macronutrient and very nearly the same micronutrient content as cow’s milk. (n=150) The substitute is a vegetable-based macronutrient substitute for milk. It consists of: almond milk (Pacific); coconut milk (Taste of Thai); and wheat gluten (King Arthur). A non-nutritive thickener (Simply Thick) made from xantham gum is added to give the consistency of whole milk. The Pacific almond milk is fortified with calcium and vitamin D. The recipe is found in Appendix 11.
5. 300 IU vitamin D supplements on schooldays (n=100). (the first month will follow the following schedule to parallel the milk “phase-in: 100 IU/day for three days; 200 IU/day for two days; 300 IU/day thereafter)
6. Larger dose vitamin D supplements once a month (n=100). The maximum daily dose of the “monthly supplement” will be 2000 IU, per Institute of Medicine upper limits for children.[Standing Committee on the Scientific Evaluation of Dietary Reference Intakes FaNB, Institute of Medicine: Dietary Reference Intakes for Calcium, Phosphorus, Magnesium, Vitamin D, and Fluoride. National Academy Press, Washington, D.C., 1997. <http://books.nap.edu/openbook.php?record_id=5776&page=285> ] The “monthly dose” will in fact be spread over several days at the start of each month. (See table 1, in Section L.3C.7 above, for the detailed schedule.)

With the exception of the unfortified milk arm (arm #3), all arms will get 14,200 IU of vitamin D over the three months. Parents of children in the unfortified milk arm will receive an equivalent amount of vitamin D supplements at the conclusion of the trial.

We will use vitamin D tablets produced by Nature Made.

Randomization method for intervention and control groups:

We will use a mix of block and individual-level randomization. Some children will receive supplements (1 of 2 supplement regimens), while other children will receive milks (one of 4 milks). Since the milk is more desirable to children than supplements, it would be unacceptable to randomize children in the same classroom to milk or supplement. However, we think it is acceptable to have one classroom in a school be a supplement classroom, and the other a milk classroom. We do this to minimize socioeconomic confounding; our primary concern here is having equivalent baseline nutritional status across intervention arms.

Ten schools will be selected using the criteria cited above. Each school will have two participating classrooms of roughly 40 children. We therefore need to randomize:

- ~200 children to one of two Supplement arms = 5 classrooms of ~40 children
- ~500 children to one of four Milk arms = 13 classrooms of ~40 children

Randomization procedure:

- Intervention schools will be randomly assigned to be one of the five ‘Mixed Intervention” or three “Milk Only Intervention” Schools by having Dr. Rich-Edwards use a random-generator program to assign classrooms.
- Classrooms within the “Mixed Intervention” schools will be randomly assigned to supplement or milk intervention in a similar fashion by Dr. Rich-Edwards. This will be done before the introductory school meetings, so that Dr. Ganmaa can streamline the consent procedure to be specific to supplement or milk classrooms.
- Block randomization: within each S or M classroom, girls and boys will be chosen by gender-stratified random assignment to receive: monthly (n=20) or daily (n=20) supplements (in each S classroom); or US milk (n=12), fortified Mongolian milk (n=12), unfortified Mongolian milk (n=10), or almond milk (n=12) (in each M classrooms).

| School | 1 | | 2 | | 3 | | 4 | | 5 | | 6 | | 7 | | 8 | | 9 | | 10 | |
| --- | --- | --- | --- | --- | --- | --- | --- | --- | --- | --- | --- | --- | --- | --- | --- | --- | --- | --- | --- | --- |
| Classroom | S1 | M1 | S2 | M2 | S3 | M3 | S4 | M4 | S5 | M5 | M6 | M7 | M8 | M9 | M10 | M11 | M12 | M13 | M14 | M15 |
|  | 20:20 | 12:12:12:10 | etc |  |  |  |  |  |  |  |  |  |  |  |  |  |  |  |  |  |

Table 2.

Training:

- Drs. Rich-Edwards and Ganmaa will work with the HSPH HSC and Mongolian ERB to design a human subjects training session appropriate for the medical student volunteers (performing the anthropometric assessments, eczema examination, and CPT attention test), the local school doctors (who will report adverse events), and phlebotomists. Since many of our collaborators are not fluent in English, they cannot undergo the standard English-based human subjects research training (such as CITI). Per the suggestion of the HSPH HSC, we will instead design a training session for them in Mongolian. To that end, we have already translated the 4-page booklet on human subjects protection in research developed by the HSPH for international studies. This is attached in Appendix 3.
- Mongolian colleagues who are fluent in English will complete the online CITI training.
- **Dr. Ganmaa and Ms. Burke will be trained by Ms. Trish Elliott in research-quality anthropometry. Ms. Elliott is the field coordinator for Boston-based Project Viva, which is currently performing anthropometry on 7 year-olds. Dr. Ganmaa and Ms. Burke, with the participation of Drs. Rich-Edwards, Frazier, and Camargo, will design a training program for the student volunteers, to include anthropometry**, eczema assessment, rickets assessment, and Tanner staging. The volunteers will be split into same-gender teams so that female volunteers will examine girls and male volunteers will examine boys.
- Dr. Ganmaa will train and supervise the milk monitors in hygienic mixing and distribution of the milk and milk substitute.

C. SAE reporting

If a child is sick, s/he will be evaluated by the local school doctor, who will grade the symptoms according to the chart with Adverse Event Grades (Table 3). The physician will report immediately to Drs. Ganmaa and Sumberzul his/her opinion of whether the illness/symptom reported is related to the trial. Dr. Ganmaa will communicate the information to Drs. Rich-Edwards and Frazier. Dr. Ganmaa will report to the ERB and Rich-Edwards will report to the HSPH HSC the details of the symptoms and the local doctor’s opinion of the likelihood that the adverse event is related to participation in the trial (“likely association with participation”, “unlikely association with participation”, “unknown association with participation”). Course of action at report of Grade 1 toxicities will be decided on a case-by-case basis. The pediatrician of the child, if s/he was not the person who did the initial evaluation, will be notified of any Grade 2 toxicities. A case report of the adverse event will be generated and submitted to the HSPH HSC and to the Ministry of Health ERB in Mongolia. We will communicate these events as quickly as possible. The only special circumstances are the time zone difference between Ulaanbaatar and Boston, which can slow communication.

Some children may experience Grade 1 or Grade 2 diarrhea, nausea, or vomiting as their systems get accustomed to the new milk diet. For that reason, we have built a ‘phase-in’ first week so that children don’t have to drink 710 ml per day from the start of the intervention.

##### Table 3: Adverse Event Grades

| Adverse Event | Grade | | | | |
| --- | --- | --- | --- | --- | --- |
|  | 0 | 1 | 2 | 3 | 4 |
| *Urticaria* *(hives, welts, wheals)* | None |  | Requiring no medication | Requiring PO or topical treatment | Requiring IV/IM medication |
| *Colitis* | None | Intermittent  abdominal pain, no change in daily functioning or diet | Abdominal pain with mucus and/or blood | Ileus or peritoneal signs |  |
| *Constipation* | None | Requiring stool softeners or dietary modification | Requiring laxatives | Obstipation |  |
| *Diarrhea* | None | Increase of <4 stools/day over pretreatment | Increase of 4-6 stools/day, or nocturnal stools | Increase of >7 stools per day or incontinence |  |
| *Dyspepsia/Heartburn* | None | Mild | Moderate | Severe |  |
| *Vomiting* | None | Mild nausea | 1 episode of vomiting in a 24 hour period | 2-5 episodes of vomiting in a 24-hour period. | Need for IV fluids |
| *Weight gain* | None | <5% | 5-<10% | 10-<20% |  |
| *Weight loss* | None | <5% | 5-<10% | 10-<20% |  |

1. Endpoints and comparisons

The primary endpoints include plasma vitamin D levels and somatotropin levels. The comparisons to be tested are numerically keyed to the table below.

The primary comparisons for the plasma vitamin D endpoints will be:

1. Post-pre intervention change in 25(OH)D for daily vs. monthly supplements (Aim 1a)

2. If there are no differences between supplement groups, then we will examine the change in

25(OH)D levels of the combined supplement groups versus the combined milk groups. If the

supplement levels are different, we will compare the change in the daily supplement group to

change in the combined fortified milk groups. (Aim 1b)

3. Change in 25(OH)D levels for combined fortified Mongolian milk group vs. unfortified

Mongolian milk (Aim 1c)

The primary comparisons for the somatotropin endpoints will be:

1. Post-intervention levels of GH and IGF-1 and IGF-1/IGFBP3 levels of Mongolian, U.S. fortified milks and the fortified milk substitute. (Aim 2b)

2. If there are no demonstrable differences in somatotropin levels between the two fortified

cow’s milks, we will combine the fortified cow’s milk groups to compare post-intervention

levels of GH, IGF-1, and IGF1/IGFBP3 levels for fortified cow’s milk vs. milk substitute.

(Aim 2a)

The secondary comparisons (eczema, asthma and respiratory infections) will be:

1. Change in eczema symptoms among children randomized to vitamin D supplements or fortified milk, in comparison to personal baseline values (Aim 3a)

2. Change in eczema symptoms in the combined fortified Mongolian milk group vs. unfortified Mongolian milk (Aim 3b)

3. Difference in frequency of respiratory infections in the combined fortified Mongolian milk group vs. unfortified Mongolian milk (Aim 4a)

4. Change in asthma control among children in the combined fortified Mongolian milk group vs. unfortified Mongolian milk (Aim 4b)

Table 4. Study comparisons

|  | **Vitamin D (post – pre)** | | | **Somatotropins (post only)** | |
| --- | --- | --- | --- | --- | --- |
| Daily supplement (n=100) | 1 | 2 |  | Unmeasured | |
| Monthly supplement (n=100) | 1 |  |  |  |  |
| Fortified U.S. milk (n=150) |  | 2 | 3 | 4 | 5 |
| Fortified Mongolian milk (n=150) |  |  |  | 4 |  |
| Fortified milk substitute (n=150) |  |  |  | 4 | 5 |
| Unfortified Mongolian milk (n=50) |  |  | 3 | Unmeasured | |

1. Statistical Analysis: Prior to formal analysis, preliminary analysis will include plotting the collected data to search for data and laboratory outliers. Although the somatotropins will not be normally distributed, the change in hormone levels is likely to be normally distributed. We will check normality of distributions and transform variables to improve normality, or use non-parametric methods of analysis if normality is not achieved.

We will then examine the following covariates to gauge the success of randomization:

- Child age in months
- Household socioeconomic status
- Baseline height, weight, and adiposity
- Baseline vitamin D and somatotropin levels

**Our main analysis tool will be a relatively simple differences-in-differences approach: we will calculate the differences between the pre-intervention and post-intervention levels within each child and then compare means between the groups.** This analysis will be accomplished using a simple t-test, as each pairwise comparison of treatment arms is of independent interest. In the event of meaningful violations of t-test assumptions, we will use the familiar nonparametric Wilcoxon test for differences between two groups. For the three-way comparison of somatotropin levels across the three milk groups, we will use an ANOVA.

1. Sample size

To determine sample sizes, we first debated the smallest clinically interesting contrast in vitamin D between arms. Since all arms (except the n=100 unfortified Mongolian milk arm) deliver the equivalent amount of vitamin D3, we anticipate small differences between them. We felt it would be as useful to definitively establish equivalency between arms as it would be to detect clinically meaningful differences; for that reason, we propose a well-powered study that can deliver tight confidence intervals. We felt that the minimum clinically meaningful difference would be 12 nmol/L (5 ng/ml). Accordingly, we designed a study to detect differences <12 nmol/L with 80% power.

In Table 5 below, we show the minimum standard deviation difference in mean 25(OH)D levels between the post-intervention arms, setting Type 1 error (alpha) to 0.05 and Type 2 error (beta) to 0.80. We used PASS statistical software for our calculations. These power calculations are conservative, in that they don’t take advantage of the pre-intervention data, which can be used to measure within-individual change. In our pilot study, we observed mean + standard deviation (s.d.) 25(OH)D levels of 43.1 + 12.0 nmol/L (17.3 + 4.8 ng/ml) at the outset of the intervention and 66.1 + 11.2 nmol/L (26.5 + 4.5 ng/ml) at the end of the one month intervention in Ulaanbaatar from May to June. These are consistent with data from adolescents in Boston winter (20.2 + 9.9 ng/ml) (Arch Pediatr Adolesc Med. 2004;158:531-537) and children in northern Spain in March (12.6 + 5.5 ng/ml).(**J Bone and Mineral Research 1998;13:544-548)** To translate the minimum detectable s.d. into clinically meaningful units of 25(OH)D, we assumed s.d.’s of 12.5 and 25 nmol/L (5 and 10 ng/ml).

Table 5.

| Contrast | N | Minimum Detectable SD units | Translation of SD into 25(OH)D units: smallest difference we could distinguish from zero with 80% power* |
| --- | --- | --- | --- |
| Monthly v. daily supplement | 100:100 | 0.40 | 5.0 to 10 nmol/L  (2.0 to 4.0 ng/ml) |
| Either supplement vs. Mongolian fortified milk; Mongolian unfortified vs. fortified milk | 100:150 | 0.36 | 4.5 to 9 nmol/L  (1.8 to 3.6 ng/ml) |
| Any supplement vs. Mongolian fortified milk | 200:150 | 0.30 | 3.7 to 7.5 nmol/L  (1.5 to 3.0 n/ml) |
| Contrast between any two fortified milks | 150:150 | 0.32 | 4.0 to 8.0 nmol/L  (1.6 to 3.2 ng/ml) |

*Assumes s.d. of 5-10 ng/mL for 25(OHD) comparisons

For the aim of examining IGF-1 and GH differences between fortified milk arms, the sample size for the primary comparisons is 150:150 (any given cow’s milk compared with milk substitute, or a contrast between two cow’s milks). As the clinical significance of changes in IGF-1 and GH within the normal range are unknown, we simply powered the study to detect changes on the order of those observed in our pilot studies. In Boston (one week intervention with macronutrient control) we observed 0.2 sd higher levels of IGF-1 and 0.4 sd higher levels of GH during the “milk week”; in Mongolia (one month intervention without macronutrient control), we observed 0.7 s.d. higher levels of IGF-1 and 0.3 s.d. higher levels of GH after the month-long milk intervention. The 150:150 contrast yields 80% power to detect differences as small as 0.3 s.d.’s, which is in the range of the contrasts we observed in the pilot studies.

1. Data storage and data security

At enrollment, the participants will be issued study ID numbers, which will be used to identify their data forms and blood samples thereafter. We will maintain the link between student identifiers and study ID in a locked file in Dr. Ganmaa’s office and in a password-protected electronic file accessible only to Dr. Ganmaa, Dr. Rich-Edwards, and designated (trained) assistants. It is necessary to maintain this linkage so that we can give the vitamin D results to the families after the samples are analyzed at the end of the study. After that point, the linkage will be destroyed.

The linkage between the participant identifiers and study ID will be destroyed on December 31, 2009, after the samples are analyzed and vitamin D results communicated to the families. We will hold on to the de-identified data for two more years to fully analyze it.

Study findings will be shared with the school community and the subjects after the study has been completed. We will return with results in the summer or fall of 2009. At a minimum, we will meet with Ministry officials and parents to communicate the study results. If we can find additional funds, we hope to hold a national conference to share the results more broadly.

At the end of the study, we plan to give the pre-intervention and post-intervention vitamin D levels to the families. Dr. Ganmaa will present the overall study results at a school meeting, and each parent will receive an envelope with their child’s vitamin D levels and an indication of whether the levels were above or below normal (50 nmol/L or 20 ng/ml). Dr. Ganmaa and the school doctor will be on hand to answer questions and make suggestions about how low levels of vitamin D deficiency can be treated and prevented with resources available in Mongolia. There are no interim results, since pre-intervention and post-intervention samples will be analyzed together at the end of the study.

LIST OF APPENDICES INCLUDED IN THE ORIGINAL APPLICATION:

1. Letter to parents describing the study
2. Script for the evening meeting with parents and children explaining the study
3. HSPH HSC Human subjects pamphlet: Mongolian version
4. Baseline questionnaire
5. Dairy Checklist
6. Classroom Behavior Questionnaire
7. EASI
8. Follow-up Visit: Respiratory Illness, Asthma, and Eczema
9. Child Study Evaluation Form
10. Parent Study Evaluation Form
11. Almond Milk Substitute Preparation
12. Package Inserts
13. Consent Form
14. Assent Form
15. Report to the Ministry of Health
16. Milk and Cancer Review
17. Foundation Proposal
18. Letter to FDA regarding IND

**Summary of changes**

**AMENDMENT #1**

1. We propose to change the milk substitute from the almond/coconut milk substitute to a milk substitute manufactured by Mead-Johnson. This substitute will be made of constituent amino acids and will be a match +10% the USDA estimates for nutrients in fortified whole cow’s milk. As the milk substitute will be flavored, Mead Johnson will also provide individual packets of flavoring that can be added to the cow’s milk arms to ensure comparability. They are also exploring producing a vitamin-D fortified flavoring that can be added to unfortified cow’s milk most widely available in Mongolia. We will provide an amendment letter with specifics as they become available.

There are several advantages of the Mead Johnson milk substitute over the almond/coconut milk we originally proposed: 1) it will be better scientifically than the almond/coconut milk because the amino acids are a closer match to cow’s milk; 2) the formula is hypoallergenic, so it is less likely to cause allergic reactions than the almond/coconut milk, and we could include children with nut or wheat allergies (who had to be excluded using the almond/coconut milk); 3) the formula will either be delivered as a powder to be mixed at the Guum plant or will be delivered in pre-mixed cans, instead of being mixed by hand by milk monitors at the school. This will improve hygiene, as well as ensure more precision in the actual intervention delivered.

Mead Johnson has indicated that they will provide these products to the study at no cost.

2. We will obtain the vitamin supplements from Tishcon Corporation. The Tishcon Corporation is a well-established company that produces vitamin and mineral supplements. Several of our colleagues have used Tishcon for their research projects involving supplements, because of the high quality control and the ability of Tishcon to produce supplements with precise vitamin quantities. In particular, Tishcon is willing to manufacture the vitamin supplements taking into account the normal degradation that happens over time to any supplement (such as might happen between the beginning and end of the 3 month trial), so that we will actually deliver the precise amount that we have proposed. Tishcon has offered to donate the supplements for this project, considering the project goals.

3. In deference to issues raised by the Mongolian ERB, we have eliminated the Tanner examination by the trained medical students. Instead, we will ask the children to complete a pencil-and-paper questionnaire (attached) which has been used by children in other studies (notably, the Growing Up Today Study) to indicate their level of pubertal development. We feel it is necessary to include some assessment of pubertal development, due to the study’s intention to measure growth. It is important to understand where in their growth spurt that children might be, and the best way we know to assess this is to have some measure of pubertal development. The ERB also suggested that the Child Evaluation Form has too many open-ended questions. We have added response choices to most of the questions.

4. We propose to supplement the Cognitive Performance Test with a 5-minute processing speed subtest of the Wechsler Intelligence Scale for Children (WISC). The test will be administered at the beginning and end of the study to see whether the nutrients provided in the milk improve children’s cognitive performance over three months.

5. We have replaced the more intensive dermatologic examination with a more simple self-report and examination of flexural dermatitis (dermatitis at skin creases, such as elbows and knees), better suited to trained medical student administration

6. Urine assay: We propose to test first morning urine void urines using the “whizpop” (pee-on-a sponge) collection kit, which we have used successfully with 6-8 year olds in Boston. The children will be given the collection kit to take home. They will test for three sequential days at the start of the study and three at the conclusion. We have added first morning urine samples because we have colleagues who report being close to developing urine-based growth hormone assays. Since growth hormone is highly variable, use of three measures at baseline and three at trial completion would provide a more stable estimate of change in growth hormone due to the intervention. Measurement of growth hormone is a primary aim of this study, so we are hopeful that the assay development will be successful. At present, we do not have a budget to cover these assays, but will seek funds if the assay development is successful.

1. Change to the consent/assent forms: We added a sentence on each form that states that “although the child's parents agree to participate in the study the child's decision will be respected”. We have made changes to reflect the use of the Mead Johnson milk substitute instead of the almond/coconut milk.

**AMENDMENT #2**

1. Mead-Johnson has completed their development of a milk substitute, and we include here its final nutritional content. There is a slight (13-23 calories per 8 ounces) difference in the calories of the milk substitute compared to the Mongolian and American milk, attributable to the slightly higher carbohydrate content of the milk substitute compared with cow’s milk. Furthermore, the US cow’s milk is somewhat more calorie dense than Mongolian cow’s milk. To maintain nutritional consistency and balance carbohydrate intake across the milk study arms, we propose to give the children low-fat crackers as part of the study protocol. We will give children enough low-fat crackers so that each arm will be comparable in terms of carbohydrates and calories.
2. The Mongolian milk producer, Guum, will also mix and package the milk substitute on a daily basis.
3. There will be no additional flavoring added to the American or Mongolian milks.
4. **In order to enroll children ages 9-11, we have elected to include 4^th^ grade classrooms in addition to the 3^rd^ grade.**

As promised, we are submitting the final version of the Food Frequency Questionnaire, now refined with the input of colleagues from the Mongolian Public Health Institute.

**AMENDMENT #3**

**In our original application, we proposed to enroll students from 10 different schools to ensure that this would allow us to enroll a total of 750 children in the study. During our September visit, we discovered that some of our targeted schools have gown rapidly as a result of increases in area populations due to immigration from the countryside. This influx of students allows us to recruit children from two schools, rather than 10. We decided to adopt this change due to budget constraints and logistic considerations.**

We have recently learned that the school vacation schedule we were originally given by the Ministry of Education is not correct for the two schools (#62 and #65) from which we will recruit students. Instead of a one week February break, the children will have vacation from January 17-25 and March 22- April 5. We had originally planned to run the intervention from January 19 through the end of March, on schooldays only. **We have regrouped with our Mongolian colleagues and the schools to change the schedule to fit the intervention in between the two vacations: from January 26-March 20, including weekends.** (During our pilot study in School #65 in 2005, the intervention was daily, including weekends).

The children in the milk arms will receive milk daily (a total of 300 IU), and children in the daily vitamin D dose arms will receive 300 IU supplements daily (instead of 5 days per week). **The intervention will still run the same total number of days.**

The children in the ‘monthly dose’ group were originally intended to receive between 1000-2000 IU over 3 days at the beginning of each month, for a total of 15000 IU over three months, the equivalent vitamin D dose as the milk and daily supplement groups. The intention was to compare the effectiveness of a once monthly supplement (easier to administer from a public health standpoint) to a once daily supplement. So that we can measure plasma vitamin D levels in the ‘monthly’ group at their nadir, we planned to draw the plasma at about 30 days after the last monthly dose.

Now that we are compressing the daily milk and daily supplement intervention into approximately 50 days instead of three months by including weekends, we also need to deliver the ‘monthly’ dose in approximately 50 days. We propose to do this by giving the children 1700-2000 IU per day for 7 days at the beginning of the trial.

In fact, the Mongolian public health system typically gives 50,000 IU once a year at the beginning of winter to infants at risk of rickets, so this strategy is familiar to them.

A daily vitamin D dose of 200-2000 IU is within the tolerable upper limit of 2000 IU per day published by the Institute of Medicine, ^1^ and well below the level at which toxicity has ever been observed (40,000 IU per day).^2^ We therefore are confident that this schedule change does not increase the risks of this study above minimal risk.

^1^Standing Committee on the Scientific Evaluation of Dietary Reference Intakes FaNB, Institute of Medicine: Dietary Reference Intakes for Calcium, Phosphorus, Magnesium, Vitamin D, and Fluoride. National Academy Press, Washington, D.C., 1997. Available online at <http://books.nap.edu/openbook.php?record_id=5776&page=285>

^2^ Vieth R, Chan P-CR, MacFarlane GD. Efficacy and safety of vitamin D3 intake exceeding the lowest observed adverse effect level. Am J Clin Nutr 2001;73:288-94.

**AMENDMENT #4**

We are adding the 34 research assistants that will be helping us to take the anthropometry measurments at the beginning and end of the protocol. They have all received CITI training. The first 17 students listed on the amendment form passed on 12/10/08. The next 18 passed CITI training on 12/17/08.

**AMENDMENT #5**

We would like to run several additional assays on the already stored blood samples from our trial. Our consent form contains the following text:

"I agree that any left over blood or urine sample can be stored for future additional research on vitamin D, infection, asthma, eczema, childhood growth and development."

The additional assays fall under the purview of the above statement because they are all relevant to infection. Only parents and children who checked yes will be included.

TGF beta 1 & 2

sIGA-

Cortisol

hsCRP

We would also like to request that Allyson Abrams be added to the study staff to do the analytic programming. Allyson's CITI certificate is attached.

**AMENDMENT #6**

We would like to run several additional assays on the already stored blood samples from our trial. Our consent form contains the following text:

"I agree that any left over blood or urine sample can be stored for future additional research on vitamin D, infection, asthma, eczema, childhood growth and development."

The additional assays fall under the purview of the above statement because they are all relevant to childhood growth and development. Only parents and children who checked yes will be included.

Estrogen metabolites panel

(E1, E2, E3, 16KE2, 16aE1, 16epiE3, 17epiE3, 3ME1, 2ME1, 4ME1, 2ME2, 4ME2, 2OHE1, 2OHE2, and 4OHE1)

**STATISTICAL PLAN AND CHANGES RELEVANT TO NEJM ANALYSIS**

**Analysis Plan for Supplementation Study and Fortification Study Vitamin D and Growth Paper**

*Goal*: to test the extent to which Vitamin D affects physical growth in children (weight, height, and BMI) in Vitamin D versus Placebo or Mongolian +D versus Mongolian regular milk.

1. Check the distribution of the outcome variables

- Run proc univariate and boxplots of each of the following for everyone:
  - Weight at Visit 1
  - Height at Visit 1
  - BMI at Visit 1
  - Weight at Visit 2
  - Height at Visit 2
  - BMI at Visit 2
  - Delta Weight
  - Delta Height
  - Delta BMI
- Repeat for each intervention arm separately
- If things are looking non-normal, try a log transformation and rerun proc univariate and boxplot
- STOP: let’s check degree of missingness in each delta
- STOP: check with Ganmaa, Lindsay and Janet to check normality and decide on any transformation

3. Re-run descriptive statistics from IGF analysis (Fortification Study only) to confirm accuracy for growth variables also evaluating combined 25OHD levels.

- Create table with all information
- (columns by treatment arm)

4. Missingness: for now, let’s do complete case analysis, unless step 2 reveals atrocious missingness, in which case we might want to create missing indicators

5. Then we can start some modeling.

- PROC REG’s for each of the deltas as dependent variables (weight, height, BMI)
  - Create indicator variables for the following interventions: Vitamin D and Placebo for Supplementation Study. Mongolian Regular, Mongolian milk + D for Fortification study. Use the Placebo and Mongolian regular arm as the referent, respectively.
  - Crude model with intervention only
  - Second model adjusted for age, school (Fortification study only), and gender
  - Third model additionally adjusted for compliance [as proportion of intervention received🡪completion
- Also model plasma 25OHD individual change against three growth delta variables (weight, height, BMI)
  - Crude model with 25OHD change only
  - Second model adjusted for intervention arm
  - Second model adjusted for age, school (Fortification study only), and gender and intervention
  - Third model additionally adjusted for compliance [as proportion of intervention received🡪completion

6. Repeat all PROC REG models for the treatment group contrasts with PROC MIXED models

- Crude model with intervention only
- Second model adjusted for age, school (Fortification study only), sex, and correlation between classrooms
- Third model additionally adjusted for compliance [as proportion of intervention received🡪completion

Models (Fortification study)

- Random effects models with 25OHD delta as the dependent variable
- Keep the multiple variance estimates for the treatment groups
- New: For all models, include an intercept and drop the centering of variables (based on a meeting with Ken)
- New: Substitute completion for compliance throughout

1. Compare each arm to Mongolian unfortified milk (Mongolian regular is reference) for Fortification Study
   1. 25OHD delta = UHT + Mon fortified + seasonal + daily
   2. 25OHD delta = UHT + Mon fortified + seasonal + daily + school + gender + age as fixed effects, and classroom as random effect
   3. 25OHD delta = UHT + Mon fortified + seasonal + daily + school + gender + age + completion as fixed effects, and classroom as random effect
2. Combine fortified milks and compare to Mongolian unfortified milk (Mongolian regular is reference; combine UHT and Mongolian fortified as one fortified group).
   1. 25OHD delta = fortified milk + seasonal + daily
   2. 25OHD delta = fortified milk + seasonal + daily + school + gender + age as fixed effects, and classroom as random effect
   3. 25OHD delta = fortified milk + seasonal + daily + school + gender + age + completion as fixed effects, and classroom as random effect
3. Compare ‘any daily’ and ‘seasonal’ to unfortified milk. (Mongolian regular is reference; combine UHT + Mongolian fortified + daily as one ‘any daily group’)
   1. 25OHD delta = anydaily + seasonal
   2. 25OHD delta = anydaily + seasonal + school + gender + age as fixed effects, and classroom as random anydaily + seasonal effect
   3. 25OHD delta = anydaily + seasonal + school + gender + age + completion as fixed effects, and classroom as random effect
4. Compare each arm to daily supplements, among groups getting vitamin D interventions (daily is referent group)
   1. 25OHD delta = UHT + Mon fortified + seasonal + Mon regular
   2. 25OHD delta = UHT + Mon fortified + seasonal + Mon regular + school + gender + age as fixed effects, and classroom as random effect
   3. 25OHD delta = UHT + Mon fortified + seasonal + Mon regular + school + gender + age + completion as fixed effects, and classroom as random effect
5. Compare “fortified milks” to daily supplements (daily is referent group; combine UHT and Mongolian fortified as one fortified group)
   1. 25OHD delta = fortified milk + seasonal + Mon regular
   2. 25OHD delta = fortified milk + seasonal + Mon regular + school + gender + age as fixed effects, and classroom as random effect
   3. 25OHD delta = fortified milk + seasonal + Mon regular + school + gender + age + completion as fixed effects, and classroom as random effect

6) Interaction models with indicator interaction and all vit D intervention groups: Exclude unfortified milk from these. Create 0/1 interaction terms between Intervention arm and v1_25OHD_below10. (the same interaction you created earlier). These should include intercept.

a. 25OHD delta = UHT + Mon fortified + seasonal + daily + school + gender + age + v1_25OHD_below10 + the interaction as fixed effects, and classroom as random effect

(here we add in the main effect of v1_25OHD_below10)

b. 25OHD delta = v1_25OHD_below10 + school + gender + age, and classroom as random effect
